# Dietary Manipulation of Amino Acids for Cancer Therapy

**DOI:** 10.3390/nu15132879

**Published:** 2023-06-25

**Authors:** Julio José Jiménez-Alonso, Miguel López-Lázaro

**Affiliations:** Department of Pharmacology, Faculty of Pharmacy, University of Seville, 41012 Sevilla, Spain; jjalonso@us.es

**Keywords:** cancer metabolism, anticancer activity, artificial diets, in vivo, mice, essential amino acids, non-essential amino acids, restriction, leucine, methionine, cysteine, arginine, serine, glutamine, asparagine

## Abstract

Cancer cells cannot proliferate and survive unless they obtain sufficient levels of the 20 proteinogenic amino acids (AAs). Unlike normal cells, cancer cells have genetic and metabolic alterations that may limit their capacity to obtain adequate levels of the 20 AAs in challenging metabolic environments. However, since normal diets provide all AAs at relatively constant levels and ratios, these potentially lethal genetic and metabolic defects are eventually harmless to cancer cells. If we temporarily replace the normal diet of cancer patients with artificial diets in which the levels of specific AAs are manipulated, cancer cells may be unable to proliferate and survive. This article reviews in vivo studies that have evaluated the antitumor activity of diets restricted in or supplemented with the 20 proteinogenic AAs, individually and in combination. It also reviews our recent studies that show that manipulating the levels of several AAs simultaneously can lead to marked survival improvements in mice with metastatic cancers.

## 1. Introduction

The first metabolic alteration of cancer cells was discovered almost one century ago by the German biochemist Otto Warburg. He observed that, unlike normal cells, cancer cells convert high amounts of glucose into lactate in the presence of normal oxygen levels [1]. This alteration in glucose metabolism, known as aerobic glycolysis or the Warburg effect, is now widely used in diagnostic imaging to trace cancers and evaluate cancer treatment responses [2,3]. The clinical use of FDG-based PET imaging has continually shown that most primary and metastatic cancers have a significant increase in glucose uptake compared to normal tissues [2,3].

Over many decades, the Warburg effect was considered to be an irrelevant oddity of cancer cells, probably because it was unknown why cancer cells used this primitive form of energy production when the availability of oxygen allows for a much more effective way of producing energy: oxidative phosphorylation. The explanation of the Warburg effect is simple when one realizes that glycolysis not only serves to produce energy, but also to produce building blocks to generate new cells [4]. Cancer cells have high glycolytic rates because the breakdown of glucose molecules generates the building blocks needed to produce many cellular components for the new cancer cells created during cell division. One cell cannot divide to produce two cells unless glucose is broken down into these building blocks. Since both glucose and oxygen are supplied together through the blood, cancer cells have no choice but to activate glycolysis in the presence of oxygen in order to proliferate. Since oxygen inhibits glycolysis (Pasteur Effect) indirectly via ATP generation, cancer cells partially uncouple oxygen utilization from ATP production to activate glycolysis in the presence of normal oxygen levels. By deviating oxygen metabolism from the route that generates ATP to the route that produces reactive oxygen species (ROS), cancer cells manage to keep sustained glycolytic rates under aerobic conditions [4,5,6]. The subsequent increased production of ROS, such as superoxide anion and hydrogen peroxide, leads to a state of increased basal oxidative stress, which represents another metabolic hallmark of cancer cells [5,7,8,9,10].

Targeting the Warburg effect for cancer therapy is difficult because cells from different normal tissues also need glucose for their survival and proliferation. However, understanding the Warburg effect is important to realize that the genetic alterations in cancer cells are insufficient for cancer cell proliferation and survival. Cancer cells also need to take glucose and other nutrients, such as amino acids (AAs), from the extracellular environment to proliferate and survive under conditions of elevated oxidative stress. Although the metabolic changes in cancer cells play an important role in carcinogenesis and cancer progression, these changes can also be exploited to develop new cancer therapies [10,11,12,13,14,15].

The altered AA metabolism of cancer cells is one of most therapeutically relevant metabolic features of cancer. Several excellent reviews have summarized the role of AA metabolism in cancer development and the potential of targeting AA metabolism for therapeutic intervention [16,17,18,19,20,21]. Briefly, cancer cells have elevated the requirements of some AAs to maintain the high biosynthetic and bioenergetic demands of cell proliferation [18]. In addition, many cancer cells are unable to synthesize sufficient levels of certain non-essential AAs (NEAAs) [21] and depend on their external supply to maintain their cellular functions. Several dietary and pharmacological interventions have been developed to target the altered AA metabolism of cancer cells [22]. For example, L-asparaginase (ASNase) is a clinically useful anticancer drug that depletes the NEAA asparagine (Asn) from the blood and selectively kills leukemia cells that cannot biosynthesize this AA [23]. Several AA-depleting enzymes and many small-molecule drugs targeting AA uptake or their metabolic pathways are currently in preclinical and clinical development [13,21]. The altered AA metabolism of cancer cells can also be targeted without drugs, through the dietary manipulation of certain AA levels [24,25,26,27].

**Figure 1:**
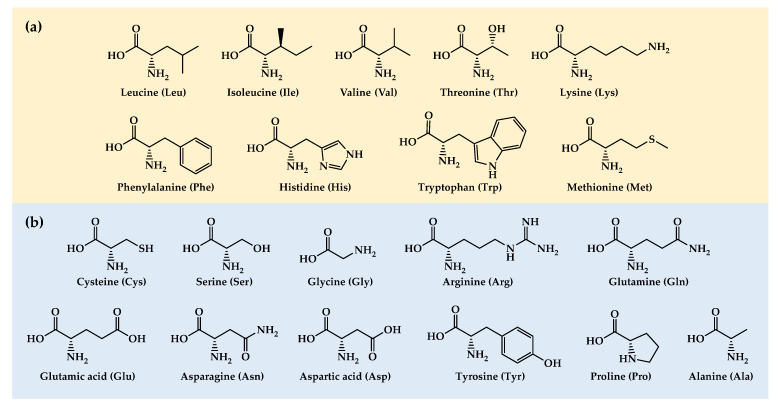
Chemical structure of proteinogenic AAs: EAAs (**a**) and NEAAs (**b**).

In this work, we review studies that have evaluated the cancer therapeutic potential of dietary AA manipulation in vivo. Since dietary proteins are the primary source of AAs for cancer cells, we first briefly review the key studies showing that protein restriction can inhibit tumor growth. Then, we review in vivo studies assessing the antitumor activity of dietary strategies based on restricting or increasing the levels of each of the 20 proteinogenic AAs, beginning with the 9 EAAs and ending with the 11 NEAAs (Figure 1). Finally, we discuss recent studies showing that manipulating the levels of several AAs simultaneously can lead to marked survival improvements in mice with different types of metastatic cancers.

## 2. Protein Restriction

Dietary protein restriction can increase life expectancy [28] and reduce the incidence of age-related diseases such as cancer [29]. It is well known that proliferating cancer cells must produce new proteins for the new cells created during cell division. Since dietary proteins provide the AAs needed to generate the proteins of the new cancer cells, it is not surprising that low-protein diets can restrict tumor growth in animal models [30]. Table 1 summarizes several studies that evaluated the effect of changing the amount and type of protein in the diet on cancer progression in mice [31,32,33,34,35,36].

A reduction in IGF-1 levels has been proposed as a key mechanism by which low-protein diets induce anticancer activity. Murine models of melanoma and breast cancer have revealed that mice fed with a low-protein diet (4% kcal protein) had reduced IFG-1 levels and a reduced tumor progression compared to those fed with a high-protein diet (18% kcal protein) [31]. Weight loss was observed in elderly mice but not in young mice. Similarly, a low-protein diet reduced the IGF-1 levels in patients aged 50–65 years and reduced the risk of cancer death, while a low-protein diet increased the mortality among elderly patients (+65 years old) [31]. This suggests that low-protein diets might induce anticancer activity in middle-aged adults but not in elderly patients. Low-protein diets are not active in all cancer types. For example, experiments on a syngeneic glioma model showed no reductions in tumor growth in animals fed with a low-protein diet (4% kcal) when compared to animals fed with a high-protein diet (18% kcal protein) [32].

Enhanced cancer immunosurveillance is another possible mechanism by which low-protein diets induce anticancer activity. A reduction in dietary proteins (17–15% protein vs. 19% protein) induced IRE1α-dependent endoplasmic reticulum (ER) stress in cancer cells, which resulted in cytokine production and improved the anticancer immune response [33]. A lower protein intake (12%) reversed this anticancer effect, which suggested that a certain level of protein intake was needed for activity [33]. However, other studies have shown that diets with a lower protein intake (7% protein vs. 21% protein) inhibited cancer progression and induced a synergistic effect when combined to anti-PD-1 immunotherapy [34]. Low-protein diets also induced anticancer activity in immunosuppressed mice, therefore suggesting that the anticancer activity of protein restriction is not necessarily mediated by the immune system [35]. For example, a low-protein diet (7% vs. 21% protein diets) reduced cancer progression in immunodeficient mice implanted with human breast and prostate cancer cells [35].

The type of protein can also modulate this cancer progression. Mice fed with a 20% plant-based protein diet showed a reduced tumor growth in xenograft and syngeneic cancer models when compared to mice fed with a 20% animal-based protein diet [35,36]. The anticancer activity of diets based on plant proteins was explained by declines in the plasma levels of IFG-1 and insulin, which decreased the activity of the IGF/AKT/mTOR pathway and led to epigenetic modifications that restricted tumor growth [35,36]. Since animal and plant proteins have different AA levels, it is important to identify which individual AAs are involved in the anticancer activity of diets based on plant proteins. Understanding the anticancer effect induced by the restriction of each AA may be useful for developing more effective diets for cancer therapy.

## 3. Essential Amino Acids

### 3.1. Leucine

Leucine (Leu) is one of the nine EAAs for humans; this means that we cannot biosynthesize it from other nutrients and we must take it from the diet. Like all 20 proteinogenic AAs, Leu is necessary for protein synthesis. Leu is also important for other cellular functions. For example, Leu is a key intracellular sensor of AAs under starvation conditions and it regulates protein turnover through mTORC1 signaling [37]. Like isoleucine (Ile) and valine (Val), Leu is a branched-chain amino acid (BCAA); these AAs can regulate the lipid metabolism in cancer cells by providing carbon skeletons for fatty-acid biosynthesis [38].

The dietary restriction of Leu can induce in vivo anticancer effects (Table 2). In 1956, Sugimura et al. [39] found that dietary deprivation of Leu for 5 days reduced the growth rate of Walker tumors in rats by 24%; however, it also induced body weight loss. In 1971, reducing the dietary levels of Leu from 0.8% to 0.1% for 3 weeks significantly reduced tumor growth in mice with breast adenocarcinomas; the tumor weights were 32 ± 10 g in mice fed with a standard AA-based diet (0.80% Leu), 38 ± 4 g for those on a 0.50% Leu diet, 32 ± 4 g for those on a 0.25% Leu diet, and 16 ± 6 g for those on a 0.10% Leu diet [40]. Body weight loss was observed in the mice fed with the 0.10% Leu diet [40]. More recent studies have shown that 14 days on a Leu-free diet combined with an autophagy inhibitor induced anticancer activity in mice with melanoma xenografts, without causing significant toxicity [41]. Only 4 days of a Leu-free diet was sufficient for inducing anticancer activity in mice with triple-negative breast cancer xenografts [42].

Mechanistically, Leu limitation restricts protein synthesis, cell division, and tumor growth. In addition, Leu restriction can reduce Leu catabolism and limit the fatty acid biosynthesis and lipogenesis in cancer cells. BCAAs catabolism plays an important role in pancreatic cancer growth by regulating lipogenesis [38]. BCAT2 and BCKDHA knockdown impaired pancreatic cancer cell proliferation in vitro and in vivo by inhibiting fatty acid synthesis [38]. Furthermore, the inhibition of BCAT1, the first enzyme in the catabolism of BCAAs, induced anticancer activity in vitro and in vivo [43,44,45,46,47,48]. Leu restriction also decreased the expression of the enzyme fatty acid synthase (FASN) [42]; FASN overexpression or palmitic acid supplementation (the product of FASN) blocked the anticancer activity of Leu restriction [42].

Since Leu restriction can reduce tumor growth, it makes sense to think that Leu supplementation may facilitate cancer progression. A study showed that a 5% Leu supplementation increased cancer growth in a syngeneic model of pancreatic cancer [49]. However, our recent studies in mice with different types of metastatic cancers indicated that supplementing Leu can increase the anticancer activity of diets deficient in other AAs [26,27]. Supplementing 2.5% Leu to several casein-based artificial diets markedly improved their anticancer activity [26,27]. Importantly, the activity of these artificial diets in mice with metastatic cancers was higher than that in the observed in mice treated with the standard therapies used for cancer patients [26,27].

**Table 2:** Results of Leu restriction/supplementation in cancer therapy in vivo.

Articles	Relevant Results in Preclinical In Vivo Cancer Models
Sugimura et al., 1959 [39]	Leu-restricted diet for 5 days reduced tumor growth in Walker cancer-bearing rats. Approximately 1–2 g/day body weight loss was observed in rats fed the Leu-restricted diet.
Theuer 1971 [40]	Dietary limitation of Leu (from 0.8% to 0.1%) for 3 weeks reduced tumor growth in mice with breast adenocarcinoma. Significant body weight loss was also observed.
Sheen et al., 2011 [41]	Leu-restricted diet for 14 days plus autophagy inhibitor (chloroquine) synergistically suppressed cancer growth in a xenograft melanoma model.
Xiao et al., 2016 [42]	Leu-restricted diet for 4 days reduced tumor growth in mice xenografted with human triple-negative breast cancer cells. Supplementation with palmitic acid and overexpression of FASN enzyme blocked the anticancer effect of Leu restriction.
Liu et al., 2014 [49]	Diet supplemented with 5% Leu enhanced tumor growth in a syngeneic pancreatic cancer model. This effect was observed in lean and overweight mice.
Jiménez-Alonso et al., 2022 [26]	Supplementation of 2.5% Leu to casein-based diets increased their anticancer activity in two syngeneic murine models of metastatic colon cancer.
Calderón-Montaño et al., 2022 [27]	Supplementation of 2.5% Leu to casein-based diets increased their anticancer activity in mice with disseminated renal cell carcinoma.

Maintaining high Leu levels may be important for preventing proteolysis, which could be beneficial in certain circumstances. Leu is a critical intracellular sensor of AAs under starvation conditions. This AA activates mTORC1 signaling and inhibits autophagy and proteasome-mediated proteolysis. Supplementing Leu may therefore prevent intracellular and extracellular proteolysis [50,51,52,53]. If muscle and liver proteolysis is not prevented, the lysis of proteins in these organs would supply any AA restricted in the diet [26,27,54]. The inhibition of proteolysis is also important in avoiding weight loss and cachexia. Cachexia is a syndrome of progressive body weight loss with reductions in skeletal muscle and fat mass [55]. Ultimately, cachexia reduces the tolerability of anticancer treatments and leads to a reduced life expectancy and quality of life [55,56]. Leu supplementation can alleviate cancer cachexia by activating mTORC1 and decreasing protein degradation [51,55,57,58]. Several preclinical studies have shown that a 3% Leu supplementation can ameliorate cancer cachexia in the Walker-256 rat model [59,60,61,62,63,64,65,66] and C26 murine model [67]. The anti-cachectic effect of Leu supplementation can be improved with fish oil supplementation [68], glutamine (Gln) supplementation [64], and aerobic physical exercise [63,64]. Evidence has suggested that a supplementation of 3% Leu is sufficient for improving cachexia [55,59,60,61,62,63,64,65,66,67,68].

### 3.2. Isoleucine

Like all proteinogenic AAs, the BCAA Ile is necessary for protein synthesis. Ile also participates in other biological processes, including lipogenesis and immune function regulation [37,38,69]. Experiments conducted several decades ago revealed that a complete dietary Ile restriction for 5 days inhibited tumor growth by 40% in Walker tumor-bearing rats; however, this force-fed intervention caused the animals to lose 1–2 g per day [39]. Dietary Ile restriction (from 0.5% to 0.05%) also resulted in tumor growth inhibition in C57BL/6 mice with BW10232 mammary carcinomas [40]. The tumor weights were 32 ± 10 g in mice fed with a standard AA-based diet (0.50% Ile), 31 ± 5 g for those on a 0.30% Ile diet, 17 ± 7 g for those on a 0.15% Ile diet, and 7 ± 3 g for those on a 0.05% Ile diet [40]. The tumors of the mice fed with the 0.15% Ile diet were significantly smaller, whereas the final tumor-free weight of the mice was relatively unaffected. Both the tumor weight and final tumor-free weight were significantly reduced in the mice fed with the 0.05% Ile diet. This means that a moderate Ile restriction was sufficient for reducing tumor growth without significantly decreasing mice body weight [40]. Mechanistically, Ile limitation restricts protein synthesis, cell division, and tumor growth. Ile restriction can also reduce Ile catabolism and limit the fatty acid biosynthesis and lipogenesis in cancer cells [38,43,44,45,46,47,48].

### 3.3. Valine

Like Leu and Ile, Val is an essential and proteinogenic BCAA. It is also involved in other cellular functions, including the regulation of lipid and glucose metabolism [37,38,70]. A dietary depletion of Val for 5 days reduced tumor growth by 41% in Walker tumor-bearing rats [39]. However, all the animals on this Val-free diet rapidly sickened and failed to survive beyond 9 days on this diet. A dietary limitation of Val (from 0.7% to 0.1%) significantly decreased tumor growth in mice with breast adenocarcinomas, but also induced body weight loss [40]. The tumor weights were 32 ± 10 g in mice fed with a standard AA-based diet (0.70% Val), 36 ± 5 g for those on a 0.40% Val diet, 25 ± 8 g for those on a 0.20% Val diet, and 16 ± 6 g for those on a 0.10% Val diet [40]. With the 0.20% Val diet, a reduction in tumor weight was achieved with little reduction in the final tumor-free weight of the mice. Reducing the Val levels further, to 0.10%, significantly reduced the tumor weight, as well as the mice tumor-free weight [40]. Mechanistically, Val limitation restricts protein synthesis, cell division, and tumor growth. Like the other two BCAAs, Val restriction can reduce Val catabolism and therefore limit the production of carbon skeletons for fatty acid biosynthesis in cancer cells [38,43,44,45,46,47,48].

### 3.4. Threonine

Threonine (Thr) is an essential and proteinogenic AA. Like other AAs, Thr catabolism can also provide amino groups for the synthesis of NEAAs and carbon skeletons for biosynthesis and energy production [37]. The force feeding of a diet lacking in Thr for 5 days reduced tumor growth by 28% in Walker tumor-bearing rats [39]. This diet caused the animals to lose between 0.2 and 1.0 g/day over an 11-day period [39]. Another study revealed that a dietary limitation of Thr for 3 weeks significantly decreased cancer growth in mice with breast adenocarcinomas [40], but also caused weight loss. The tumor weights were 32 ± 10 g in mice fed with a standard AA-based diet (0.50% Thr), 37 ± 6 g for those on a 0.30% Thr diet, 30 ± 7 g for those on a 0.15% Thr diet, and 15 ± 5 g for those on a 0.05% Thr diet [40]. In a group of mice not inoculated with the breast adenocarcinoma cells, feeding them the 0.05% Thr diet for three weeks produced a 31% weight loss [40]. Thr limitation restricts protein synthesis, cell division, and tumor growth.

### 3.5. Lysine

Lys is an essential and proteinogenic AA, whose deficiency can trigger severe malnutrition [37,71]. Lys is also used for carnitine production and participates in protein methylation, acetylation, ubiquitination, and glycosylation [37]. The anticancer activity of Lys restriction was evaluated 80 years ago in mice with spontaneous breast cancer [72]. The author of this research first devised a Lys-deficient diet suitable for human consumption (palatable, adequate in calories, minerals, and vitamins, and sufficient for keeping nitrogen balance). After observing in two healthy humans that nitrogen equilibrium could be maintained with this diet, he obtained and reproduced a strain of mice characterized by a high incidence of spontaneous mammary carcinomas. The mice that developed tumors were fed with the Lys-deficient diet. The diet inhibited the growth rate of the tumors, but also the rate of normal growth in the mice. These inhibitory effects were abolished upon the addition of Lys, therefore indicating that Lys was essential for both normal and malignant growth. When the Lys-deficient diet was fed to them for several weeks, the antitumor effect wore off and the tumors resumed rapid growth. In addition, the inhibitory effect was either not apparent or very short when the Lys-deficient diet was started in mice with tumors that had reached an advanced stage of growth. The author concluded that the therapeutic potential of the Lys-deficient diet was low [72]. In 1959, the force feeding of a diet lacking in Lys for 5 days did not reduce tumor growth in Walker tumor-bearing rats [39]. In 1971, a Lys limitation (from 0.6% to 0.15%) did not significantly reduce tumor growth in C57BL mice with BW10232 mammary carcinomas [40]. The tumor weights were 36 ± 13 g in mice fed with a standard AA-based diet (0.90% Lys), 36 ± 6 g for those on a 0.60% Lys diet, 36 ± 6 g for those on a 0.30% Lys diet, and 31 ± 8 g for those on a 0.15% Lys diet [40].

### 3.6. Phenylalanine

Phenylalanine (Phe) is an essential and proteinogenic AA with an aromatic group in its structure. Phe can be used to synthesize tyrosine (Tyr), a proteinogenic NEAA that produces important molecules such as catecholamines (dopamine, epinephrine, and norepinephrine) and melanin [37]. Dietary Phe limitation is used in people with phenylketonuria, an inborn disease caused by the inactivity of the enzyme phenylalanine hydroxylase, which converts Phe into Tyr; the accumulation of Phe can lead to seizures and intellectual disability [73].

In 1959, the force feeding of a diet lacking in Phe for 5 days was found to reduce tumor growth by 15% in Walker tumor-bearing rats [39]. Several years later, a Phe-deficient diet (0.12% Phe) was reported to reduce tumor growth by 23% in C57L/J mice with BW7756 hepatomas and 32% in C3H/HeJ mice with C3HBA mammary adenocarcinomas [74]. In combination with ρ-fluorophenylalanine (a metabolic analog of Phe), the Phe-deficient diet reduced tumor growth by 94% in the BW7756 hepatomas and 42% in the C3HBA mammary adenocarcinomas [74].

Since Phe is a precursor of Tyr, the dual restriction of Phe and Tyr was evaluated in several studies conducted between the 1960s and the early 2000s. In 1966, a diet with 0.12% Phe and 0.06% Tyr was found to reduce the growth of melanoma (but not of sarcoma) in mice [75]. In 1971, a dietary restriction of Phe and Tyr reduced tumor growth in C57BL mice with BW10232 mammary carcinomas [40]. The tumor weights were 41 ± 9 g in mice fed with a standard AA-based diet (0.60% Phe + 0.30% Tyr), 35 ± 10 g for those on a 0.40% Phe + 0.20% Tyr diet, 29 ± 6 g for those on a 0.20% Phe + 0.10% Tyr diet, and 11 ± 5 g for those on a 0.10% Phe + 0.05% Tyr diet [40]. In a group of mice not inoculated with the breast cancer cells, feeding them the 0.10% Phe + 0.05% Tyr diet for three weeks produced a 21% weight loss [40]. The dietary limitation of Phe and Tyr also showed anticancer activity in mice with breast cancer but not fibrosarcoma [76]. A diet with 0.08% Phe and 0.04% Tyr reduced the metastatic potential of cancer cells in several in vivo models, including melanoma, lung, and hepatocarcinoma [77,78]. In vitro experiments using different types of cancer cells support the in vivo anticancer activity of Phe and Tyr limitation [79,80,81,82,83,84,85,86].

In humans, several case reports have shown reductions in the tumor bulk and regression of lymph nodes in patients with malignant melanoma, Hodgkins lymphoma, and cancer of the uterus [87,88]. The stabilization of choroidal malignant melanoma has also been reported [89]. In 1985, no tumor responses were observed in three patients with disseminated malignant melanoma who received a low Phe/Tyr diet for two months [90]. In 2002, three patients with metastatic melanoma and three patients with metastatic breast cancer agreed to consume a low-protein diet providing approximately 10 mg/kg Phe/Tyr per day; the diet was based on several fixed products and complemented with different foods [91]. A possible decline in the rate of disease progression was observed in one patient with metastatic melanoma; this patient had a prognosis of 8 weeks upon recruitment, but survived a further 7 months after stopping the low Phe/Tyr diet [91]. All the patients of this pilot study experienced side effects such as increases in anxiety and depression [91].

### 3.7. Histidine

Histidine (His) is an aromatic EAA required for protein synthesis. This AA is involved in other cellular functions, including the synthesis of histamine and carnitine [37,92]. The force feeding of a diet lacking in His for 5 days reduced tumor growth by 19% in the Walker rat model [39]. More recently, the dietary limitation of His was found to selectively limit the growth of MYC-dependent neural tumors in a *Drosophila* model [93]. In contrast, supplementing His can activate His catabolism, which consumes tetrahydrofolate and increases the anticancer activity of methotrexate by reducing the tetrahydrofolate cellular pool [94]. This study found that an administration of His (injection of 18.4 mg His) significantly increased the anticancer activity of methotrexate in mice xenografted with human leukemia cells [94].

### 3.8. Tryptophan

Although tryptophan (Trp) is the least abundant EAA in the diet, it is necessary for protein synthesis and the production of a variety of biologically active compounds, including serotonin, melatonin, and niacin (a component of NAD and NADP) [37,95]. In addition, Trp and its catabolic derivatives modulate the immune function and play key roles in autoimmune diseases and antitumor immunity [95,96].

In 1959, a total Trp restriction for 5 days inhibited tumor growth by 19% in Walker tumor-bearing rats, with moderate weight loss in the animals [39]. In 1971, a Trp limitation (from 0.10% to 0.02%) reduced tumor growth in C57BL/6 mice with BW10232 mammary carcinomas [40]. The tumor weights were 33 ± 6 g in mice fed with a standard AA-based diet (0.15% Trp), 33 ± 13 g for those on a 0.10% Trp diet, 31 ± 10 g for those on a 0.05% Trp diet, and 16 ± 8 g for those on a 0.02% Trp diet [40]. The mice fed with the 0.02% Trp diet lost 28% of their weight in 3 weeks [40]. A moderate dietary limitation of Trp (0.05%) did not show anticancer activity in C3H mice bearing mammary adenocarcinomas [97].

Recent research on Trp and cancer therapy has focused on a catabolic pathway known as the kynurenine (Kyn) pathway, in which Trp is catabolized into Kyn by the enzymes indoleamine-2,3-dioxygenase 1 and 2 (IDO1/2) and tryptophan-2,3-dioxygenase (TDO2) [96]. Tumor and myeloid cells in the tumor microenvironment are known to metabolize Trp to Kyn [95]. A drop in Trp levels and increases in the levels of the metabolites of the Kyn pathway can lead to an immunosuppressive state that supports cancer survival [95,96,98,99]. For example, the antitumor activity of anti-PD1 immunotherapy was reduced in mice fed with a low Trp diet [100]. Trp limitation may therefore facilitate cancer progression by impairing cancer immunosurveillance. Several IDO1 and TDO2 inhibitors have enhanced the anticancer activity of checkpoint inhibitors in preclinical studies, and some of them have entered clinical trials, including epacadostat (phase I to III), BMS-986205 (phase I–II), indoximod (phase II), and navoximod (phase I) [95,96,101]. Unfortunately, epacadostat and other compounds have yielded disappointing clinical results. Epacadostat plus pembrolizumab did not improve progression-free survival and overall survival compared to pembrolizumab alone in a phase III clinical trial with 706 melanoma patients [102]. More research is needed to fully understand the relevance of Trp metabolism in cancer progression and immunity.

### 3.9. Methionine

Methionine (Met) is an essential and proteinogenic AA that contains a sulfur atom in its structure. Met is the precursor of S-adenosyl methionine (SAM), which is a methyl donor involved in DNA methylation and epigenetics. Met also produces Cys through the irreversible transsulfuration pathway, which, in turn, produces several sulfur-containing molecules with important cellular roles, including glutathione (GSH), hydrogen sulfide (H_2_S), and taurine (Tau) [24,37,103,104].

Dietary Met restriction has shown anticancer activity in numerous preclinical studies [26,27,34,39,40,105,106,107,108,109,110,111,112,113,114,115,116,117,118,119,120,121,122,123,124,125]. A dietary Met depletion (0%) induced anticancer activity in rats [39,121,122,123,124] and mice [105,106,107,108,109,110,111,125] with different types of cancer. The force feeding of a diet lacking in Met for 5 days reduced tumor growth by 39% in the Walker rat model, but this diet caused the animals to lose l–2 g weight/day [39]. Met restriction (from 0.60% to 0.10%) reduced tumor growth in C57BL mice with BW10232 mammary carcinomas [40]. The tumor weights were 36 ± 13 g in mice fed with a standard AA-based diet (0.90% Met; 0.2% Cys), 33 ± 11 g for those on a 0.60% Met diet, 30 ± 6 g for those on a 0.40% Met diet, 29 ± 7 g for those on a 0.20% Met diet, and 16 ± 7 g for those on a 0.10% Met diet [40]. The mice fed with the 0.10% Met diet lost 10% of their initial weight in 3 weeks [40]. Several studies have suggested that Met intake can be reduced to 0.12% without causing significant protein loss or noticeable toxicities in healthy animals [126,127,128]. Limiting Met intake to 0.17–0.12% did not significantly decrease the body weight of mice with different types of cancer [112,113,114,115,116]. Since Met is necessary for biosynthesizing Cys, the dietary levels of Cys can condition the dietary requirements of Met. A dietary restriction of Met can increase the antitumor effects of a variety of drugs, including 5-fluorouracil [106,114,117,121,124], anti-PD-1 immunotherapy [34,118], vincristine [122], cisplatin [105], lexatumimab (TNFα receptor agonist) [109], auranofin (TXNRDs inhibitor) [110], ethionine (Met analog) [123], and radiotherapy [114]. Table 3 shows representative studies assessing the in vivo anticancer activity of Met restriction.

Mechanistically, Met limitation restricts protein synthesis, cell division, and tumor growth. Met restriction can also reduce the cellular levels of the methyl donor SAM, which may alter the DNA methylation and epigenetics in dividing cancer cells. Met restriction can also compromise the biosynthesis of polyamines, which are involved in several key processes of cell growth and survival, including the maintenance of protein and nucleic acid synthesis, the stabilization of the chromatin structure, and protection from oxidative damage [130]. Since Met is necessary for synthesizing Cys, Met restriction can lead to Cys restriction when the dietary intake of Cys is low. In this case, Met restriction can reduce Cys and GSH (Glu-Cys-Gly) levels, which may lead to the accumulation of cytotoxic concentrations of ROS in cancer cells. Accordingly, evidence has suggested that Cys supplementation can reduce the anticancer activity of Met restriction [131,132], and many studies have restricted or eliminated Cys in the diet to increase the anticancer activity of this Met restriction [26,27,34,40,111,113,120,121,122,133]. It has been proposed that cancer cells are more vulnerable than normal cells to Met restriction because cancer cells may be unable to recycle Met from homocysteine (HCys) or SAM through the Met salvage pathways [24]. Normal cells can proliferate under Met-restricted conditions if supplied with HCys, while cancer cells cannot obtain sufficient Met from HCys [134,135,136]. This vulnerability may be explained by defects in the enzymes involved in the Met salvage pathways [137,138]. Furthermore, some cancer cells use HCys to synthesize Cys, therefore limiting their ability to recycle Met [139].

The role of Met in antitumor immunity is complex. Met supplementation can improve immune function because this AA is highly consumed by immune T-cells. Accordingly, intratumoral/intraperitoneal Met administration has improved antitumor immunity and increased the anticancer activity of checkpoint inhibitors in several syngeneic cancer models [140]. On the other hand, some studies have shown that a dietary Met limitation can improve antitumor immunity and increase the activity of anti-PD-1 immunotherapies [34,118].

Dietary Met restriction has been evaluated in cancer patients in phase I clinical trials. In 1995, 14 patients with preoperative gastric cancer were randomly divided into two groups: 5-fluorouracil plus total parenteral nutrition lacking Met, or 5-fluorouracil plus total parenteral nutrition with Met [141]. The combination of 5-fluoruracil plus Met restriction showed a lower tumor burden and thymidylate synthase activity compared to 5-fluoruracil alone [141]. In 2002, eight patients with solid cancers received a Met-restricted diet for approximately 17 weeks (range 8–39 weeks) [142]. This Met-restricted diet was safe and tolerable for the patients [142]. In the late 2000s, two small clinical trials were conducted with a total of 29 patients with melanomas and 3 patients with gliomas [143,144], who received an intermittent Met-free diet plus cystemustine [144] or nitrosourea [143]. The treatment was well tolerated, but little benefit was observed in terms of patient survival. In 2010, a phase I trial was conducted with 11 colon cancer patients who received three cycles of a Met-free diet for 3 consecutive days plus FOLFOX6 chemotherapy [145]. The plasma Met levels were reduced by 58% on the first day and the treatment was well tolerated, but little benefit was observed [145]. Recently, after observing that Met restriction produced therapeutic responses in patient-derived xenograft models of chemotherapy-resistant RAS-driven colorectal cancer in mice, the authors conducted a feeding study on humans that revealed that Met restriction induced changes in their systemic metabolism that were similar to those obtained in mice [114].

Pharmacological approaches based on an enzymatic depletion of Met support the idea that Met restriction has potential for cancer therapy. Methioninase (METase) was the first enzyme developed to deplete Met. The injection of this recombinant bacterial enzyme can deplete Met from the plasma and induce anticancer activity [146]. In mice, each administration completely depletes Met for 8 h, showing anticancer activity against xenograft and syngeneic cancer models [147,148,149,150,151,152]. METase can also deplete Met for 8 h in primates [153]. However, since METase has a short half-life (immune clearance) and its continuous administration could trigger anaphylactic reactions [153], the enzyme was pegylated to minimize its immunogenicity. PEGylated-METase extended this half-life and prevents anaphylactic reactions in primates [154]. Surprisingly, an oral administration of METase, which removes Met from the gastrointestinal tract, was as effective as an intraperitoneal administration in lowering serum Met levels and inducing anticancer activity in several murine cancer models [155,156,157,158,159,160,161,162]. Other human recombinant enzymes have recently been developed [163,164]. These human recombinant enzymes elude the immunogenic problems of the original METase and have demonstrated in vivo anticancer activity against neuroblastoma [163] and prostate cancer [164]. Met enzymatic depletion has been combined with several anticancer drugs. Enzymatic Met depletion has shown synergistic effects with 5-fluorouracil [149], cisplatin [148], temozolomide [150,151], nitrosourea [150], and doxorubicin [152]. Oral METase has also shown a synergistic effect in combination with various anticancer drugs [155,156,157,158,159,160,161,162]. The pharmacokinetics and safety of METase have also been evaluated in patients. Two small phase I clinical trials conducted during the late 1990s showed that METase reduced the plasma levels of Met without causing evident toxicity [165,166]. Recently, two case reports have shown that an oral administration of METase plus a low-Met diet achieved stable long-term disease in a patient with locally recurrent rectal cancer [167] and in a patient with stage IV pancreatic cancer treated with FOLFIRINOX [168].

## 4. Non-Essential Amino Acids

### 4.1. Cysteine

**Figure 2:**
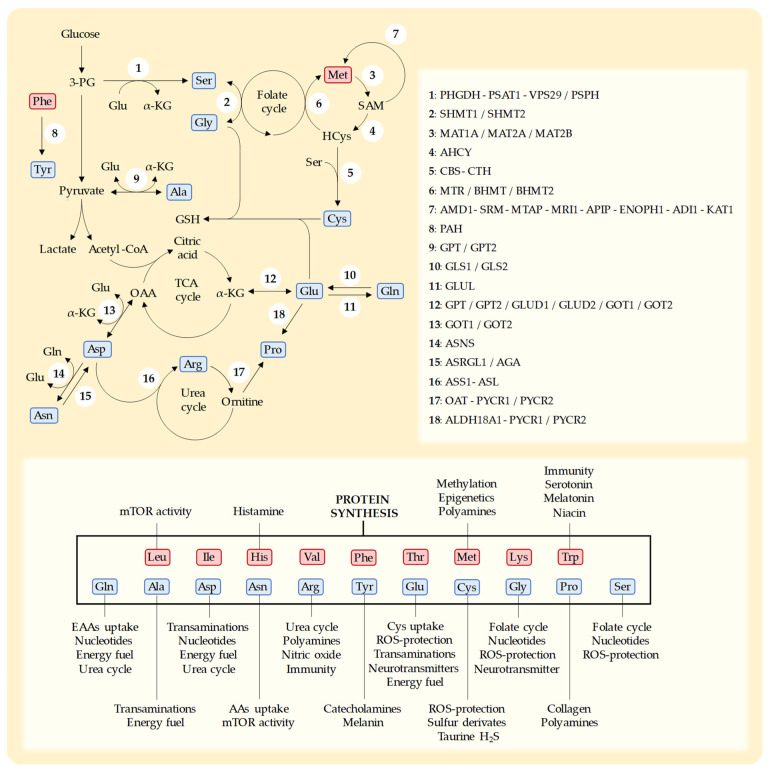
Schematic representation of key metabolic pathways for the biosynthesis of NEAAs, the enzymes in each pathway, and the main functions of each AA [54,173]. NEAAs are represented in blue and EAAs in red. Leucine (Leu), isoleucine (Ile), histidine (His), valine (Val), phenylalanine (Phe), threonine (Thr), methionine (Met), lysine (Lys), tryptophan (Trp), glutamine (Gln), alanine (Ala), aspartate (Asp), asparagine (Asn), arginine (Arg), tyrosine (Tyr), glutamate (Glu), cysteine (Cys), glycine (Gly), proline (Pro), and serine (Ser). 3-phospho-D-glycerate (3-PG), S-adenosylmethionine (SAM), homocysteine (HCys), glutathione (GSH), α-ketoglutarate (α-KGlu), tricarboxylic acid cycle (TCA), oxaloacetate (OAA), reactive oxygen species (ROS). D-3-phosphoglycerate dehydrogenase (PHGDH), phosphoserine aminotransferase-1 (PSAT1), vacuolar protein sorting-associated protein-29 (VPS29), phosphoserine phosphatase (PSPH), serine hydroxymethyltransferase-1 (SHMT1), serine hydroxymethyltransferase-2 (SHMT2), S-adenosylmethionine synthase isoform type-1 (MAT1A), S-adenosylmethionine synthetase isoform type-2 (MAT2A), methionine adenosyltransferase 2 subunit beta (MAT2B), adenosylhomocysteinase (AHCY), cystathionine β-synthase (CBS), cystathionine γ-lyase (CTH), methionine synthase (MTR), betaine-homocysteine methyltransferase (BHMT), betaine-homocysteine methyltransferase-2 (BHMT2), S-adenosylmethionine decarboxylase (AMD1), spermidine synthase (SRM), 5′-methylthioadenosine phosphorylase (MTAP), methylthioribose-1-phosphate isomerase (MRI1), methylthioribulose 1-phosphate dehydratase (APIP), enolase-phosphatase (ENOPH1), 1,2-dihydroxy-3-keto-5-methylthiopentene dioxygenase (ADI1), 2-oxo-4-methylthiobutanoate aminotransferase (KYAT1), phenylalanine hydroxylase (PAH), alanine aminotransferase-1 (GPT), alanine aminotransferase-2 (GPT2), glutaminase-1 (GLS1), glutaminase-2 (GLS2), glutamine synthetase (GLUL), glutamate dehydrogenase-1 (GLUD1), glutamate dehydrogenase-2 (GLUD2), aspartate aminotransferase-1 (GOT1), aspartate aminotransferase-2 (GOT2), asparagine synthetase (ASNS), asparaginase (ASRGL1), aspartylglucosaminidase (AGA), argininosuccinate synthase (ASS1), argininosuccinate lyase (ASL), ornithine aminotransferase (OAT), pyrroline-5-carboxylate reductase-1 (PYCR1), pyrroline-5-carboxylate reductase-2 (PYCR2), and δ-1-pyrroline-5-carboxylate synthase (ALDH18A1). Enzymes that participate in consecutive steps in a metabolic pathway are separated by “-” and enzymes that catalyze the same step in a metabolic pathway are separated by “/”.

Cys is a sulfur-containing NEAA with multiple cellular roles [169]. Humans can biosynthesize Cys from the EAA Met through the transsulfuration pathway [24,169,170] (Figure 2). In addition to being necessary for protein synthesis, Cys is essential for the production of a variety of sulfur-containing molecules with important biological roles [170]. These include the iron–sulfur clusters found in enzymes of the electron transport chain (ETC) [171], coenzyme A, and thioredoxins [172]. Cys also produces taurine [37] and hydrogen sulfide (H_2_S) [37] and is the rate-limiting AA for the production of the tripeptide GSH (Glu-Cys-Gly) [169,170]. GSH is essential for protecting cells against the toxic effects ROS [169].

The importance of Cys in tumor growth was first reported in 1936 [133]. In this study, Voegtlin et al. observed that a diet deficient in Cys/Met reduced tumor growth in mice with spontaneous breast cancer, and the addition of Cys abruptly stimulated tumor growth [133]. Since Met can produce Cys, Met has usually been restricted in many studies evaluating the anticancer activity of Cys depletion/restriction [26,27,34,40,111,113,120,121,122,133]. Intravenous parenteral nutrition with double Cys/Met restriction showed anticancer activity in rats with sarcoma [121,122] and inhibited the cancer proliferation in mice xenografted with human glioma cells [111]. A dietary restriction of Cys and Met also showed anticancer activity in a spontaneous mouse model of breast cancer [40] and transgenic prostate cancer model [34]. We recently showed that an artificial diet restricted in Cys and Met (formulated with 6% casein, 5% Gln, and 2.5% Leu) induced a marked anticancer activity in two metastatic colon cancer models; Cys supplementation blocked its anticancer activity [26]. Although limiting Met levels can increase the effect of Cys restriction, anticancer activity has been observed with Cys-restricted diets with normal levels of Met, for example in animal models of colon cancer [174,175] and gliomas [176]. We recently found that a diet lacking in six NEAAs (including Cys), with normal Met levels (0.6%), showed marked anticancer activity in mice with disseminated renal cell carcinomas; the anticancer activity of this diet was reduced by Cys supplementation [27]. However, supplementing Cys under certain conditions may be important for the anticancer activity of an anticancer diet; we recently observed that supplementing 0.2% Cys in an inactive casein-based diet (restricted in Cys) markedly improved the survival of mice with disseminated renal cell carcinomas [27]. Cys restriction may therefore have a positive or negative effect on the anticancer activity of a diet, depending on the levels of its other dietary components [27]. Table 4 shows representative studies assessing the in vivo anticancer activity of Cys restriction.

Mechanistically, Cys restriction may induce anticancer activity by reducing the capacity of cancer cells to eliminate ROS. Cancer cells produce high levels of ROS, which may accumulate and produce cell death [8]. Cancer cells rely on GSH to reduce these ROS levels [24]. A dietary Cys restriction can decrease Cys plasma levels [176], reduce GSH biosynthesis [175,176], and increase the ROS levels in cancer cells [174,175,176]. Cys restriction may also lead to the accumulation of cytotoxic levels of ROS in cancer cells by interfering with the activity of the polyamine pathway, which cancer cells use for ROS protection [174].

Since Cys is necessary for immune cells, Cys restriction may reduce the ability of the immune system to eliminate cancer cells. Cys is essential for T-cell activation and function [177]. High CysS plasma levels have been associated with a higher probability of response to immune checkpoint inhibitors in patients with lung cancer [178,179]. However, the negative effect of Cys restriction on the immune antitumor response is controversial, because other studies have suggested that Cys restriction can increase the antitumor immune response [34,180].

Pharmacological approaches based on an enzymatic depletion of Cys and inhibition of Cys transporters support the idea that Cys restriction has potential for cancer therapy. These pharmacological interventions have been useful for understanding the possible mechanisms by which Cys restriction induces in vivo anticancer effects. In 2017, an optimized human cyst(e)inase enzyme was able to reduce the Cys and CysS plasma levels in mice and primates without causing toxicity [181]. Cyst(e)inase has shown anticancer activity in mouse models of a variety of cancers, including prostate, breast, chronic lymphocytic leukemia, pancreas, lung, renal, melanoma, and ovarian cancer [180,181,182,183,184,185,186]. Cyst(e)inase administration increases ROS levels, depletes the intracellular levels of GSH, and triggers ferroptosis in cancer cells [181,182,183,184,185,186]. Ferroptosis is a form of iron-dependent cell death triggered by lipid peroxidation. GPX4, which prevents lipid peroxidation, needs GSH as a cofactor for its activity [170,187,188]. Cys depletion can lead to lipid peroxidation and trigger ferroptotic cancer cell death.

The pharmacological inhibition of the xCT antiporter (SLC7A11), which imports exogenous CysS and exports glutamate (Glu), suggests that cancer cells depend on external Cys supply for their survival. Due to the oxidizing conditions of the extracellular environment, most extracellular Cys is in the form of CysS (the oxidized dimer of Cys) [189]. An inhibition of the xCT antiporter decreases the intracellular Cys levels, causes a loss in antioxidant protection, and induces ferroptosis [189]. Several drugs are known to inhibit xCT antiporter activity, including sulfasalazine, sorafenib, erastin, imidazole ketone erastin (IKE), and HG106 [189]. These drugs, some of them approved for clinical use, induce anticancer activity in a variety of cancer models [190,191,192,193,194,195,196,197,198,199,200]. Sulfasalazine, which is used for the treatment of rheumatic arthritis, has been evaluated in several clinical studies. Sulfasalazine, at a maximum dose of 6 g/day, did not induce a clinical response in 10 patients with gliomas [201]. In a dose-escalation study, sulfasalazine reduced intratumoral GSH levels and CD44-positive cancer stem cells in patients with gastric cancer [202]. In a clinical trial conducted with eight patients with CD44-positive gastric cancer who received sulfasalazine plus cisplatin, only one patient achieved stable disease for 4 months [203]. A phase I/II clinical trial with patients with glioblastomas revealed that the addition of sulfasalazine to temozolomide plus radiotherapy intervention did not increase their overall survival and progression-free survival [204].

### 4.2. Serine

Serine (Ser) is synthesized from 3-phosphoglycerate (glucose metabolite) and Glu (nitrogen donor) through the de novo Ser synthesis pathway [205]. In addition to being a proteinogenic AA, Ser plays an important role in one-carbon metabolism [205,206,207]. Ser is the main source of carbon units in the folate cycle, which is mainly used for the synthesis of purines and pyrimidines and the conversion of HCys into Met. Ser is also used to produce Gly and provides the carbon skeleton for the synthesis of Cys through the transsulfuration pathway. It also has other important functions, such as the production of certain lipids, including ceramide and phosphatidylserine [205,206,207].

Ser and Gly are easily interconverted by SHMT1/2 enzymes [207]. Therefore, Gly is usually restricted in most dietary studies evaluating the anticancer activity of Ser limitation. Dietary Ser/Gly can reduce the Ser and Gly levels in plasma [208] and tumors [209]. Although both AAs can be synthesized by human cells, cancer cells may depend on an external supply of these AAs to keep their high proliferative demands. Cancer cells may also have mutations (e.g., in *p53*) that increase their dependency on these AAs. A dietary double restriction of Ser/Gly has shown anticancer activity against a variety of cancers in mice [208,209,210,211,212,213,214,215,216,217,218,219,220,221]. These studies are summarized in Table 5.

Mechanistically, dietary Ser/Gly restriction can induce anticancer activity by restricting two important building blocks in biosynthesis. The new cancer cells created during tumor growth need new proteins, nucleic acids, and specific lipids; these processes require the synthesis or acquisition of sufficient levels of these two AAs. For example, dietary Ser/Gly restriction induced anticancer activity in a colon cancer xenograft model by altering the biosynthesis of sphingolipids [212]. Dietary Ser/Gly restriction may also induce anticancer activity by increasing the cellular levels of ROS in cancer cells. Ser is needed to synthesize Gly and Cys, which are necessary for producing the antioxidant tripeptide GSH (Glu-Cys-Gly). Ser restriction caused oxidative stress in p53-deficient cancer cells, and dietary Ser/Gly restriction induced anticancer activity in mice [214]. Combinations of Ser–Gly-restricted diets with other pro-oxidant treatments have shown synergistic anticancer responses in murine cancer models [213,218].

Some mutations found in cancer cells can increase their susceptibility to dietary Ser/Gly restriction, including mutations involved in the synthesis of Ser/Gly or some mutations in p53. However, an overexpression of the enzymes involved in the biosynthesis of these AAs may compromise the anticancer activity of dietary Ser/Gly restriction. For example, many cancer cells overexpress PHGDH, the first enzyme involved in the synthesis of Ser (Figure 2) [206]. Certain p53 mutations and the activation of KRAS, MYCN, NRF2, and MDM2 can induce the overexpression of the enzymes involved in Ser biosynthesis, conferring resistance to dietary Ser/Gly restriction [24,208,211,215,222]. As expected, reduced expressions of PHGDH and PSAT1 sensitized cancer cells to Ser–Gly-restricted diets [211,221]. A low expression of PHGDH has been observed in platinum-resistant ovarian cancer cells, making this subtype of ovarian cancer vulnerable to Ser/Gly dietary restriction [210]. A combination of dietary Ser/Gly restriction with an inhibitor of PHGDH (PH755) showed anticancer activity in colon cancer [217].

The importance of Ser and Gly for cancer cell proliferation and survival is supported by studies showing that a pharmacological inhibition of the enzymes involved in the Ser/Gly biosynthesis pathway induces anticancer effects. The PHGDH inhibitors NTC-503 [219,223] and CBR-5884 [224] induced antiproliferative effects, but lacked selectivity for PHGDH. The PHGDH inhibitor PH755 showed a higher selectivity and induced anticancer activity in vitro and in vivo [212,217,225]. Small-molecule dual SHMT1/2 inhibitors also showed anticancer activity against B-cell and T-cell lymphoblastic leukemia [226,227].

In immunogenic tumors, however, dietary Ser/Gly restriction may reduce the ability of immune cells to eliminate cancer cells. For example, Ser restriction can impair the expansion of T cells in vivo, probably because Ser supplies Gly and one-carbon units for de novo nucleotide biosynthesis in proliferating T cells [228]. We recently observed in mice with disseminated renal cell carcinomas that a diet with both Ser and Gly was better than the same diet without Ser and Gly; the untreated mice lived for 30.3 ± 1.3 days, mice fed with the diet without Ser/Gly lived for 40.3 ± 2.0 days, and mice fed with the diet with Ser/Gly lived for 54.7 ± 7.8 days [27].

### 4.3. Glycine

Gly is an NEAA that can be synthetized from Ser. Gly is essential for protein synthesis. Collagen, which is the most abundant protein in the human body (30–40% of total body protein), contains approximately 33% of Gly [229]. This AA also acts as an inhibitory neurotransmitter [37]. Gly can also be used for the synthesis of the antioxidant tripeptide GSH, Ser, purines, creatine, and heme group [37]. Evidence has suggested that rapidly growing cancer cells have a high Gly dependency [230].

As discussed in the previous section, Ser and Gly are easily interconverted by the SHMT1/2 enzymes [207]. Therefore, in most studies evaluating the anticancer activity of dietary Gly restriction, Ser has also been restricted. Gly/Ser restriction has shown anticancer activity in different types of cancer [208,209,210,211,212,213,214,215,216,217,218,219,220,221,222]. Table 5 shows representative studies assessing the in vivo anticancer activity of Ser/Gly restriction. We recently observed that an artificial diet lacking several NEAAs (Ser, Cys, Tyr, Pro, Asn, and Glu) markedly improved the survival of mice with disseminated renal cell carcinomas; Gly elimination, however, reduced the activity of this diet. These studies suggest that restricting Gly can have a positive or negative effect on the anticancer activity of a diet, depending on the levels of its other dietary components [27].

### 4.4. Arginine

Arginine (Arg) is an NEAA used for protein synthesis. It also participates in many other biological processes, including the synthesis of nitric oxide, creatinine, ornithine, agmatine, and polyamines [37,231]. It also plays a key role in the urea cycle [37]. Normal cells can synthesize Arg from citrulline and aspartate (Asp) through ASS1 (argininosuccinate synthase 1) and ASL (argininosuccinate lyase) in the urea cycle.

The anticancer potential of Arg restriction was evaluated in 1959 by Sugimura et al. [39]. In this study, 5 days of an Arg-free diet reduced the growth of Walker tumors in rats, and no weight loss was observed after 11 days on the Arg-free diet [39]. In the early 1990s, an Arg-free diet showed anticancer effects in mouse models of skin carcinogenesis [232] and colon cancer [233]; the Arg-free diet inhibited tumor growth and Arg supplementation stimulated tumor growth [233]. More recently, a dietary Arg restriction reduced cancer growth in a xenograft model of ASS1-deficient breast cancer [234]. Arg-restricted diets also suppress the cancer growth in colon cancer [235], prostate cancer [236], and liver cancer [237] xenografts. On the other hand, since adequate levels of Arg are important for T-cell proliferation, the dietary supplementation of Arg has been found to improve T-cell function and induce anticancer immunity in murine cancer models alone and in combination with other anticancer drugs [238,239,240,241,242]. Table 6 summarizes studies assessing the in vivo anticancer activity of dietary Arg restriction and supplementation.

Arg-free diets can decrease the plasma levels of Arg in healthy volunteers. An Arg-free diet taken for 6 days reduced Arg plasma levels by approximately 20–40% [243]. In another study, 4 weeks of a dietary restriction of Arg and other precursors of Arg (Asp, Pro, and Glu) significantly decreased Arg plasma levels without causing side effects [244].

Mechanistically, dietary Arg deprivation may induce selective anticancer activity because many cancer cells express low levels of ASS1, which is involved in the synthesis of Arg. The downregulation of ASS1 facilitates cancer cell proliferation by increasing the aspartate availability for pyrimidine biosynthesis [245]. In addition, downregulating the expression of ASS1 under acidic or hypoxic environments provides cancer cells with a survival advantage [246]. However, ASS1-deficient cancer cells rely on the external supply of Arg for their survival, which may explain why Arg deprivation induces anticancer activity [246].

The importance of Arg for cancer cell proliferation and survival has been supported by numerous studies that have shown that a pharmacological depletion of Arg levels with Arg-depleting enzymes induces anticancer activity. Two different enzymes are currently under clinical development: ADI-PEG20 (pegylated arginine deiminase) and PEG-BCT-100 (pegylated recombinant human arginase 1). These enzymes have shown anticancer activity in a wide variety of cancers, including melanoma, hepatocarcinoma, and glioblastoma [231,234,247,248,249]. Eight phase I-II clinical trials have been completed or are ongoing for PEG-BCT-100 [250]. In patients with hepatocellular carcinomas, this drug was well tolerated and showed anticancer activity alone [251,252,253] and in combination with chemotherapy (capecitabine plus oxaliplatin) [254]. PEG-BCT-100 also showed anticancer activity in melanoma and prostate cancer patients [255], and induced complete remission in an immunotherapy-resistant melanoma patient with an absent expression of the enzymes involved in the synthesis of Arg [256]. ADI-PEG20 has received more clinical attention, with 30 completed or ongoing clinical trials, three of them in phase III [257]. ADI-PEG20 monotherapy was well tolerated and safe in most clinical trials [258,259,260,261,262,263,264,265,266]. Combinations of ADI-PEG20 with other anticancer drugs are also being studied in phase I-II clinical trials [267,268,269,270,271,272,273]. In a phase III clinical trial with more than 600 patients with hepatocellular carcinomas, ADI-PEG20 monotherapy did not show significant improvements in their overall survival and progression-free survival [265]. More research is needed to elucidate these possible mechanisms of resistance, as well as the potential benefit of the combination of this enzyme with other anticancer therapies.

As occurs with other AAs, Arg restriction may have a negative impact on immunogenic cancers. Some cancer cells create an immunosuppressive microenvironment by converting myeloid cells into M2 macrophages or myeloid-derived suppressive cells [274]. These immunosuppressive cells express arginase, which hydrolyzes Arg to ornithine and urea, therefore reducing the Arg levels in the tumor microenvironment [274]. Arg is essential for T-cell proliferation and the expression of arginase can disrupt antitumor immunity [241,274,275]. Accordingly, arginase inhibitors (which increase Arg levels) have shown anticancer activity [276,277,278,279] and have reached phase I-II clinical trials [280,281]. A dietary supplementation of Arg can improve immune function and induce anticancer activity in murine cancer models alone and in combination with other anticancer drugs [238,239,240,241,242]. However, in a recent double-blind clinical trial with 65 colorectal cancer patients, 10 g/day of Arg supplementation did not prevent immunosuppression compared to a placebo [282]. In summary, since Arg is important for both cancer cells and immune cells, patients with Arg-auxotrophic tumors may benefit from Arg-restriction therapies, while patients with immunogenic cancers may benefit from Arg supplementation.

### 4.5. Glutamine

Gln is a non-essential proteinogenic AA that can be considered as essential under certain conditions [283]. It is the most abundant AA in human plasma and tissues and is involved in many biological processes [284]. It participates in the transport and detoxification of ammonia in the urea cycle, helping to maintain the pH balance [285,286]. Gln is the main source of nitrogen atoms for the biosynthesis of nucleotides (pyrimidines and purines) and NEAAs (Glu, Asn, Ala, Asp, Ser, Pro, and citrulline) [285]. Gln also mediates the cellular uptake of certain EAAs; for example, LAT1 imports the EAA Leu while simultaneously exporting Gln [287]. Importantly, Gln catabolism is used as a key energy source in highly proliferating cells, such as intestinal cells, immune cells, and cancer cells [286]. Gln is deaminated in two steps by GLS and glutamate dehydrogenase (GDH), yielding Glu and α-ketoglutarate (αKG); the latter can enter the tricarboxylic acid (TCA) cycle and eventually produce ATP [286]. Gln indirectly participates in maintaining cellular redox balance. Gln catabolism generates Glu, which is used for the synthesis of GSH and the uptake of CysS through the antiporter xCT [288]. Gln is a versatile biosynthetic substrate to supply carbon and nitrogen atoms for the generation of the key precursors for biosynthesis and cell proliferation [289].

Proliferating cancer cells have a high Gln demand. Cancer cells obtain high Gln levels by increasing their biosynthesis or by obtaining it from the extracellular environment [285]. The increased Gln uptake of cancer cells has been associated with lower plasma levels of Gln in patients with several types of cancer [290,291]. The increased Gln uptake by tumors is actually being studied for diagnostic purposes with PET imaging using 18F-(2S,4R)-4-fluoroglutamine [292,293,294,295]. The increased Gln uptake of cancer cells is related to their high expression of ASCT2 (SLC1A5) [296,297,298,299,300]; this chief Gln transporter is upregulated by the oncogenes MYC and KRAS [301,302]. Once Gln is inside the cell, Gln catabolism serves to supply the TCA cycle, support lipogenesis, biosynthesize NEAAs and nucleotides, and help to maintain high GSH levels [285,303]. The GLS1 isoform, which catalyzes Gln deamination to generate Glu, is upregulated by the oncogene MYC [301]. GLS1 is upregulated in many cancer types and its overexpression is associated with a poor prognosis [304,305,306,307,308,309]. The conversion of Glu into αKG is also enhanced in many cancer cells, probably because MYC, KRAS, and PI3KCA expression upregulate the enzymes GLUD1, GOT1, GOT2, and GPT2 [310,311,312]. Gln may become an essential AA for cancer cells driven by oncogenic MYC, KRAS, and PIK3CA [310].

Limiting Gln levels and targeting Gln acquisition and utilization have been studied as possible anticancer strategies. Few studies have evaluated the in vivo anticancer activity of diets deficient in Gln. In 2017, the dietary restriction of Gln was found to induce anticancer activity in vitro and in vivo in a p73-expressing medulloblastoma xenograft model [313]. The Gln-restricted diet increased mice survival and also showed a synergistic effect with cisplatin. Although the only difference between the control and experimental diets was the presence/restriction of Gln, both diets also lacked Glu, Ala, Asn, Asp, and Pro. This diet reduced the Gln and Glu levels in the cerebellum and cerebrospinal fluid of the mice [313]. In another study, a Gln- and Glu-free diet significantly decreased Gln plasma levels and impaired disease progression in mice with Notch1-expressing leukemia [314].

Most anticancer strategies targeting the altered Gln metabolism of cancer cells have focused on the pharmacological inhibition of Gln acquisition and utilization [285]. These include the inhibition of GLS1 with inhibitors such as CB-839 (telaglenastat) [315], BPTES [316], and C.968 [317,318]. CB-839, which is orally bioavailable, has been tested in clinical trials. There are at least 21 completed or ongoing phase I-II clinical trials, 8 of which have been completed [319]. In general, CB-839 was safe and well tolerated by cancer patients [320,321,322,323,324,325,326]. In most of the completed clinical trials, CB-839 was combined with other anticancer drugs [285]. Its benefit for cancer progression has been modest so far [285]. There are other experimental anticancer drugs targeting Gln metabolism. The inhibition of Gln uptake by V-9302, an inhibitor of the ASCT2 transporter, induced anticancer activity in murine cancer models [327]. JHU083, which is a prodrug of the Gln antagonist DON [328], is selectively activated in the tumor microenvironment and disrupts cancer cell metabolism while improving T-cell anticancer responses. This compound induced marked anticancer activity alone and in combination with immunotherapies in several murine cancer models [328,329,330,331,332,333]. The off-target effects of some anticancer drugs can also impact Gln metabolism. For example, the Asn-depleting enzyme L-asparaginase (ASNase), which is used against leukemia and relies on external supply of Asn, can also deplete Gln; this effect may explain its anticancer activity in a murine leukemia model resistant to Asn depletion [334,335].

Gln restriction may cause toxicity because this AA is necessary for non-malignant proliferating cells [336,337]. A dietary restriction of Gln induced small intestine mucosal atrophy and muscle weight loss in rats [338]. Oral Gln supplementation has been shown to ameliorate mucosal damage (mucositis, stomatitis, pharyngitis, esophagitis, and enteritis) induced by chemotherapy and radiotherapy in randomized clinical trials with cancer patients [339,340,341]. Gln plays a key role in the cellular uptake of Leu [287], and its supplementation could enhance the beneficial effects of Leu on cancer cachexia [55].

A recent report showed that Gln supplementation induced in vivo anticancer activity in a transgenic melanoma model and sensitized tumors to a BRAF inhibitor via epigenetic reprogramming [342]. The authors observed that a diet containing very high levels of Gln (20%) increased the concentrations of Gln and αKG in tumors, without increasing the other biosynthetic intermediates necessary for cell proliferation. The increase in αKG concentration led to the hypomethylation of H3K4me3, thereby suppressing the epigenetically activated oncogenic pathways in melanoma [342]. Our recent investigation revealed that supplementing specific artificial diets with Gln can increase their anticancer activity in mice with metastatic cancers; most of our active diets contained 5–6% Gln in their composition [26,27,343]. Therefore, although Gln plays a key role in cancer cell metabolism, anticancer activity can be achieved by both restricting and supplementing this AA.

### 4.6. Glutamate

Glu is an NEAA closely related to Gln. This AA is used in protein synthesis and has many other cellular functions. Glu is as a nitrogen donor for transaminases [37]. It is used in the synthesis of many NEAAs, including Ala, Asp, Ser, Pro, and Gln (Figure 2) [54]. Transaminases and glutamic dehydrogenase (GDH) can convert Glu into αKG, which can be used to fuel the TCA cycle for energy production. In the brain, Glu is an excitatory neurotransmitter and can also be used for the synthesis of the inhibitory neurotransmitter γ-aminobutyric acid (GABA) [37]. Glu participates in ROS protection by allowing CysS uptake by the xCT antiporter, and by directly participating in the synthesis of the tripeptide GSH [288].

Although Glu supports cancer cell proliferation and survival, the anticancer activity of dietary Glu restriction has not been extensively studied, probably because Glu can be easily obtained from Gln, Asp, and Ala, and is also produced in the degradation pathways of many AAs, including Leu, Ile, Val, Lys, Phe, His, Tyr, and Pro. We recently observed that a diet lacking in six NEAAs (including Glu) induced marked anticancer activity in mice with disseminated renal cell carcinomas; supplementing Glu in this diet markedly reduced its anticancer activity [27]. Glu is particularly abundant in the brain. Glioblastomas and brain tumors use this AA for energy production [344] and nucleotide biosynthesis [345]. As mentioned previously, dietary Gln restriction showed anticancer activity in a medulloblastoma xenograft model, and a significant drop in the levels of both Gln and Glu was observed in the cerebellum and cerebrospinal fluid of mice treated with this diet [313]. The activity of this diet may therefore be mediated, at least in part, by a reduction in the levels of Glu. Similarly, a Gln- and Glu-restricted diet achieved anticancer responses in mice with Notch1-expressing leukemia [314]. Accordingly, as mentioned previously, the enzyme GLS produces Glu from Gln, and several GLS inhibitors have shown in vivo anticancer activity, including CB-839 (telaglenastat) [315], BPTES [316], and C.968 [317,318]. CB-839 has been evaluated in clinical trials [319,320,321,322,323,324,325,326]; however, its clinical benefit has been moderate [285].

### 4.7. Asparagine

Asn is an NEAA that can be synthesized from Asp by the enzyme ASNase. Asn is needed for protein synthesis, but the importance of Asn in other cellular processes is less understood [20]. Asn can modulate mTORC1 activity and serve as an exchange molecule for the uptake of other AAs (e.g., Ser, Arg, and His), and the maintenance of intracellular Asn levels seems to be critical for cancer cell growth [346].

Asn is commonly used to exemplify the relevance of NEAA restriction in cancer therapy, because the Asn-depleting enzyme ASNase is a useful drug for patients with acute lymphoblastic leukemia (ALL) and acute lymphoblastic lymphoma (ALLy). ASNase is an enzyme from *E. coli* that deaminates Asn to Asp and ammonium; its intravenous administration quickly depletes the Asn from serum and cells [347]. ALL cells usually rely on external Asn for their survival, and the depletion of Asn with ASNase leads to apoptosis in leukemia cells [348]. ASNase is pegylated (PEG-ASNase) to extend its half-life and reduce the immunogenicity of the enzyme. Nowadays, ASNase is included in most chemotherapy regimens for pediatric ALL and ALLy, achieving high survival rates [23]. The efficacy of ASNase is generally correlated with the expression of ASNS in leukemia cells [349,350]; this enzyme allows the synthesis of Asn from Asp. However, in some cases, ASNS expression after ASNase has not been associated with resistance to treatment [351]. ASNase also has Gln-depleting activity, which may participate in the anticancer activity of this enzyme [334,335]. ASNase treatment can produce adverse effects attributed to Gln-depleting activity, ammonia production, and the development of immunogenicity against the enzyme [352]. Antibody neutralization and a subsequent inactivation of ASNase are key causes of treatment failure [23]. As an alternative for patients with hypersensitivity reactions and silent neutralization to *E. coli* ASNase, *Erwinia* ASNase can be used as a second-line treatment [353]. Although ASNase is considered inactive against most solid tumors [354], growing evidence has suggested that ASNase has anticancer activity in several types of solid tumors in preclinical animal models [216,355,356,357,358,359,360].

Dietary Asn restriction induces in vivo anticancer activity [216,357,360] and may serve as an alternative to treatment with ASNase. Limiting Asn availability via an ASNS knockdown, treatment with ASNase, or dietary Asn restriction reduced the number of lung metastases in an orthotopic triple-negative breast cancer model, whereas increased dietary Asn or enforced ASNS expression promoted metastatic progression [357]. The serum Asn levels were proportional to the Asn content in each diet (0% Asn in the restricted diet, 0.6% in the standard diet, and 4% in the supplemented diet) [357]. Another study revealed that dietary Asn restriction reduced tumor growth in KEAP1 mutant cancer cells in vivo [216]. Cancer cells with KEAP1/NRF1 mutations display a high endogenous oxidative stress response, dependent on the external supply of several AAs, including Asn [216]. ASNase showed more anticancer activity than dietary Asn restriction, and the combination of both enzymatic and dietary restriction showed the same activity as ASNase alone [216]. Recently, a combination of dietary Asn restriction with the electron transport chain (ETC) inhibitors metformin or IACS-010759 induced anticancer activity in xenograft and transgenic lung cancer models [360]. Similar results were obtained with ASNase in combination with metformin [360]. Supplementing 0.6% or 4% Asn restored tumor growth [360]. Mechanistically, an inhibition of the ETC with metformin limits the Asp availability for Asn synthesis during Asn-restricted conditions [360].

### 4.8. Aspartate

In addition to its role in protein synthesis, Asp participates in the synthesis of purines, pyrimidines, Asn, and Arg [37]. It also plays a role in the urea cycle, the malate-Asp shuttle, and transamination reactions [37]. Due to its role in the synthesis of nucleotides, Asp is crucial for proliferating cancer cells.

Although Asp can become a limiting factor for tumor growth, the antitumor activity of dietary Asp deprivation has not been evaluated individually, probably because this AA can be easily obtained from Glu and OAA through GOT1/2 (AST) transaminases (Figure 2). Since these enzymes are expressed in many tissues, including the liver, a dietary Asp restriction would not result in a systemic Asp restriction.

Recent evidence has indicated that Asp is an endogenous metabolic limitation for tumor growth. Asp has a poor cell permeability, which prevents its environmental acquisition by tumor cells [361]. Cancer cells synthetize Asp from the OAA originated in the TCA cycle; this process requires ETC activity to consume NADH and allow OAA synthesis from malate [362,363]. Therefore, cancer cells rely on the TCA cycle and ETC to obtain Asp for proliferation and other processes such as Asn biosynthesis. As mentioned before, this has been exploited to increase the anticancer activity of dietary Asn restriction. The combination of an Asn-restricted diet with an ETC inhibitor suppressed tumor progression in transgenic and xenograft lung cancer models [360]. An inhibition of the ETC (metformin or IACS-010759) reduced the intracellular Asp available for Asn synthesis under Asn-restricted conditions [360]. Some cancer cells overcome their dependence on Asp production from the TCA cycle by expressing the Asp/Glu carrier 1 (AGC1 or SLC1A3), allowing Asp to uptake into cells [364]. The expression of SLC1A3 in cancer cells provided resistance to ETC inhibition in xenograft lung cancer models [364]. Gln catabolism can feed the TCA cycle and therefore Asp synthesis; the deletion of SLC1A3 synergized with CB-839 (GLS inhibitor) in a syngeneic model of lung cancer [365].

### 4.9. Tyrosine

Tyr is an aromatic NEAA that can be obtained from the EAA Phe. In addition to its role in protein synthesis, Tyr is necessary for producing catecholamines (dopamine, epinephrine, and norepinephrine) and melanin [37].

Since Phe is a precursor of Tyr, both AAs are usually restricted simultaneously in most cancer studies. As shown in the Phenylalanine section (Section 3.6), a dual restriction of Phe and Tyr has been evaluated in animal studies and cancer patients with several positive results. We recently found that a diet lacking in six NEAAs (Tyr, Cys, Ser, Pro, Asn, and Glu) markedly improved the survival of mice with disseminated renal cell carcinomas. Adding Tyr to this AA-manipulated diet did not decrease its anticancer activity, suggesting that restricting Tyr was not essential for achieving anticancer activity in this cancer model [27].

### 4.10. Alanine

Ala is a proteinogenic NEAA with other important metabolic functions. It is involved in transamination reactions and the glucose-alanine cycle (Cahill cycle). Ala can be easily converted into pyruvate by GPT1/2 transaminases [37]; pyruvate is a carbon source for energy production, fatty acid biosynthesis, and gluconeogenesis [37,366].

The antitumor activity of dietary Ala deprivation has not been evaluated independently of other AAs, probably because Ala can be easily obtained from Glu and pyruvate through GPT1/2 transaminases (Figure 2). Since these enzymes are expressed in many tissues, including the liver, a dietary Ala restriction would not result in a systemic Ala restriction.

Recent evidence has suggested that Ala is a critical substrate for pancreatic cancer cells. Pancreatic cancer cells obtain Ala from stroma-associated pancreatic stellate cells by upregulating the SLC38A2 transporter. Pancreatic cancer cells then deaminate Ala to obtain pyruvate and support the TCA cycle and biosynthesis of NEAAs and lipids. Pyruvate derived from Ala can actually outcompete glucose and Gln-derived carbon skeletons for these processes [367,368].

### 4.11. Proline

Pro is a proteinogenic NEAA that can be synthesized from Glu or ornithine [369] (Figure 2). Pro can be used for the synthesis of Arg, Glu, and polyamines, and participates in wound healing and the immune response [37,370]. Like Gly, Pro is a major building block for the synthesis of collagen [37]. Collagen is the main Pro storage in the human body [369], and some cancer cells, such as pancreatic cancer cells, can use extracellular collagen to obtain Pro under conditions of nutrient deprivation [371].

Dietary Pro restriction inhibited tumor growth in mice xenografted with PC-9 lung cancer cells, but not in mice with PaTu-8902 pancreatic cancer cells [372]. Mechanistically, Pro starvation induced endoplasmic reticulum stress in cancer cells with a hyperactivation of mTORC1-mediated 4EBP1 signaling [372]. A diet restricted in several NEAAs (including Pro) showed anticancer activity in mice inoculated with B16F10 melanoma cells; the diet was initiated one week before the inoculation of the cancer cells [373]. We recently observed that the addition of Pro to a diet lacking in six NEAAs (Tyr, Cys, Ser, Pro, Asn, and Glu) did not block its anticancer activity in mice with disseminated renal cell carcinomas [27].

The pharmacological treatment of the enzymes involved in Pro metabolism suggests that this AA plays an important role in the metabolism of cancer cells. Pro catabolism is used by cancer cells to generate Glu and αKG, which can fuel the TCA cycle [369]. Since PRODH is a key enzyme in Pro catabolism, several PRODH inhibitors have been developed to target cancer cells. N-propargyl-glycine is a suicide inhibitor of PRODH that has shown anticancer activity in breast cancer xenografts [374]. The inhibition of PRODH with L-tetrahydro-2-furoic acid (L-THFA) decreased growth and metastases in breast and lung cancer models [375,376]. On the other hand, an overexpression of the enzymes involved in Pro biosynthesis, such as PYCR1 and OAT (Figure 2), can facilitate cancer progression, and their pharmacological inhibition can suppress cancer growth in vivo [377,378,379,380,381].

## 5. Manipulation of Multiple Amino Acids Simultaneously

Since the metabolic routes of many AAs are interconnected (Figure 2), the cellular requirements of specific AAs are probably influenced by the levels of other AAs. Manipulating several AAs simultaneously may therefore be more therapeutically useful than restricting AAs individually. As discussed in the previous sections, several pairs of AAs have usually been restricted together. Phe is the precursor of Tyr, and several studies have shown in vivo anticancer activity when both AAs were restricted together [40,75,76,77,78]. Similarly, since Met can be used to synthesize Cys, a dietary restriction of both AAs has induced in vivo anticancer activity in different cancer types [26,27,34,40,111,113,120,121,122,133]. The NEAAs Ser and Gly can be easily interconverted by SHMT1/2 enzymes (Figure 2), and the simultaneous restriction of both AAs has induced anticancer activity in several murine cancer models [208,209,210,211,212,213,214,215,216,217,218,219,220,221,222].

We recently reported that the manipulation of multiple AAs simultaneously induced marked anticancer activities in mice with different types of metastatic cancers [26,27,119,343]. We screened 18 artificial diets for anticancer activity in mice with disseminated renal cell carcinomas and observed that mice fed with a diet lacking in six AAs (Ser, Cys, Tyr, Pro, Asn, and Glu) lived longer than mice treated with sunitinib or anti-PD-1 immunotherapy (which are standard therapies for patients with metastatic renal cell carcinomas). Controlling the levels of several AAs (e.g., Cys, Met, and Leu) and lipids was important for the anticancer activity of the diets [27]. We also tested several artificial diets in mice with metastatic colon cancer and compared their activity with that of capecitabine, which is a first-line treatment for patients with this disease. Mice fed with a diet lacking in 10 AAs (all NEEAAs except Gln), or a diet with 6% casein, 5% Gln, and 2.5% Leu, lived longer than untreated mice; several mice survived the treatment. The casein-based diet was better than several cycles of capecitabine in two animal models; the models were established by injecting CT26.WT murine colon cancer cells in the peritoneum (peritoneal dissemination) or tail vein (pulmonary metastases) of immunocompetent BALB/cAnNRj mice. We found that Cys supplementation blocked the activity of both diets, but Cys restriction was insufficient for activity [26]. We also reported that the survival of mice with metastatic triple-negative breast cancer (TNBC) could be markedly increased by replacing their normal diet with artificial diets in which the levels of AAs and lipids were strongly manipulated. AA manipulation led to modest improvements in mice survival when the levels or lipids were normal. Reducing the lipid levels to 1% markedly improved the activity of several diets with different AA contents. Mice fed with the artificial diets as monotherapy lived longer than mice treated with the first-line drugs doxorubicin and capecitabine. An artificial diet without 10 NEAAs, with reduced levels of EAAs and 1% lipids, improved the survival not only of mice with TNBC, but also of mice with other types of metastatic cancers [343]. Two additional diets with altered levels of sulfur AAs also improved the survival of mice with metastatic colon cancer, ovarian cancer, and renal cell carcinomas [119]. These data suggest that the dietary manipulation of multiple AAs simultaneously has therapeutic potential for patients with metastatic cancers. Based on these results, we are currently testing the safety and efficacy of one of our artificial diets as monotherapy for patients with different types of metastatic cancers. A synthetic meal replacement without NEAAs has also been tested in patients with prostate cancer (NCT04389918).

## 6. Discussion

Two decades ago, few cancer scientists considered metabolism as a relevant area of cancer research, probably because it was assumed that the accumulation of mutations in a cell was sufficient for malignant transformation, cell division, and tumor growth. The explanation of the Warburg effect revealed that the genetic alterations in cancer cells are insufficient for cell proliferation and tumor growth. Cell proliferation requires that the dividing cell takes nutrients, such as glucose and certain AAs, from the extracellular environment. If you culture any type of cancer cell in PBS (which lacks glucose and AAs), the cell will not proliferate, no matter what type of mutations it has. Cells must uptake glucose and AAs to produce the building blocks for creating new cells. The widespread clinical use of FDG-based PET imaging reminds us that most cancers have increased glucose uptake compared to normal tissues [2,3]. Unfortunately, glucose deprivation is not a feasible therapeutic strategy for cancer patients, because normal tissues also need glucose for their survival and proliferation. Severe hypoglycemia, which may occur in patients with type I diabetes receiving insulin, can actually be lethal if untreated. Depriving cells of certain AAs, however, can selectively kill cancer cells [382] and also be useful for patients with specific cancers [23]. Today, AA metabolism is considered to be a therapeutically relevant area of cancer research.

It is important to stress that selectivity is the key feature of an effective anticancer treatment. Cancer patients need therapies able to eliminate their malignant cells without significantly affecting their normal cells. The available anticancer drugs can kill cancer cells through a variety of mechanisms of action, but they also target normal cells at similar concentrations. The consequence of this limited selectivity is that patients cannot be treated with the drug doses needed to eliminate their malignant cells, because these doses would also kill their normal cells and be fatal. Cancer patients receive tolerable doses instead of effective doses, which are generally insufficient for eradicating the cancer cells and curing the disease. This means that, when searching for clinically relevant therapies based on AA manipulation, the key is not to find the most toxic strategies for cancer cells. The key is to find strategies able to eliminate cancer cells without significantly affecting normal cells.

Restricting NEAAs may be more clinically relevant than restricting EAAs. A complete deprivation of EAAs would be toxic to both cancer cells and normal cells, because human cells cannot synthesize these AAs. However, normal cells can synthesize NEAAs, while cancer cells may be unable to obtain all of them because of their mutated genomes. This difference may confer selectivity. In vitro experiments have revealed that restricting NEAAs, individually and simultaneously, can induce selective anticancer effects [26,27,382] However, these experiments should be interpreted cautiously, because the metabolic environments of cells growing in vitro and in vivo are extremely different. For example, the low AA concentrations tested in most in vitro experiments are difficult to achieve in the systemic circulation, because the liver and muscles provide AAs to ensure sufficient plasma levels. Since in vitro data are difficult to extrapolate to an in vivo situation, this review article is focused on in vivo studies that have evaluated the antitumor activity of diets restricted or supplemented with the 20 proteinogenic AAs, individually and in combination.

Restricting protein levels is the simplest strategy for reducing all AAs simultaneously. Several studies have shown that low-protein diets reduce tumor growth in animal models [30,31,32,33,34,35]. Reducing the levels of plant proteins induced higher antitumor activities than reducing the levels of animal proteins, probably because each type of protein contains different ratios of AAs [34,35]. Reducing protein levels, however, does not exploit the full therapeutic potential of AA manipulation. Since each protein has a constant AA ratio, changing these protein levels does not allow an alteration of the levels of specific AAs while keeping normal levels of others. Using mixtures of AAs, alone or in combination with proteins, can drastically change AA ratios and may increase the potential of dietary AA manipulation for cancer therapy.

Diets lacking any EAA are lethal if taken continuously. However, these diets may induce anticancer activity if taken temporarily. Reducing EAAs to certain levels, rather than eliminating them, may also be therapeutically useful. Several decades ago, researchers started to evaluate in rodents the antitumor activity of diets lacking each EAA and diets with reduced levels of each EAA [39,40,72]. For most EAAs, the antitumor activity of the diets was linked to marked reductions in body weights; the deprivation of EAAs was toxic for both cancer tissues and normal tissues. However, reducing some EAAs to certain levels (e.g., Ile, Met, and Phe) inhibited tumor growth without significantly affecting body weight. For example, a moderate restriction of Ile in the diet (0.15%) was sufficient for reducing tumor growth without significantly decreasing body weight [40]. More recent experiments have revealed that limiting Met intake to 0.17–0.12% induced anticancer activity without causing significant weight loss in mice [112,113,114,115,116]. Many cancer cells have defects in the Met salvage pathways [134,135,136,137,138,139], which may limit their ability to recycle Met under low-Met conditions [24]. In cancer patients, several case reports and pilot clinical studies have shown that a dietary restriction of Phe/Tyr or Met only induced mild anticancer effects (see Section 3.6 and Section 3.9).

Diets lacking in NEAAs are not lethal because our cells can synthesize these AAs. Some cancer cells, however, may have lost the ability to synthesize particular NEAAs due to mutations or low expressions of the enzymes involved in NEAA biosynthesis (Figure 2). For example, some cancer cells are known to depend on extracellular Asn or Arg for their survival. A restriction of Asn with the anticancer drug ASNase has been used in patients with leukemia for decades [23,349,350]. Dietary Asn restriction has also shown anticancer activity in preclinical models of solid tumors [216,357,360]. Similarly, some cancer cells lack the Arg-producing enzyme ASS1 and depend on exogenous Arg for their survival [231]. Dietary Arg restriction induces anticancer activity in preclinical models [232,233,234,235,236,237]. An enzymatic depletion of Arg has also shown preclinical anticancer activity and reached clinical trials [250,258,259,260,261,262,263,264,265,266]. In a phase III clinical trial with more than 600 patients with hepatocellular carcinomas, the Arg-depleting enzyme ADI-PEG20 did not improve their progression-free survival, although a mild overall survival benefit associated with prolonged Arg depletion was noted [265].

Limiting individual AAs in the diet has shown anticancer activity in animal models. However, the available clinical data show that this strategy may be insufficient for improving the survival rates achieved with the standard pharmacological treatments. The average rate of a successful translation from animal models to clinical cancer trials is generally low; possible explanations for and solutions to this problem have been proposed [383,384,385]. To date, Asn is the only AA whose restriction has been approved for patients with specific cancers. Since the cellular requirements of specific AAs are influenced by the levels of others, manipulating several AAs simultaneously may be more clinically useful than restricting AAs individually. In addition, it is important to realize that increasing, rather than reducing, the levels of particular AAs may improve the efficacy of diets based on AA manipulation. For example, if we restrict or eliminate some AAs in a diet, we may need to keep sufficient levels of Leu to avoid muscle and liver proteolysis; otherwise, the lysis of proteins in these organs would supply the AAs restricted in the diet. Supplementing a diet with particular AAs may also be important for creating a permissive environment for antitumor immunity. Cells of the immune system must proliferate to mount an efficient immune response, and immune cells need to acquire sufficient levels of certain AAs (e.g., Trp, Met, Cys, Ser, Gly, Arg, Gln, and Asn) to proliferate. For example, Arg supplementation has improved antitumor immunity alone and in combination with immunotherapies [238,239,240,241,242]. Limiting AAs may therefore be a double-edged sword in cancer therapy. Reducing AA levels may inhibit cancer cell proliferation, but may also inhibit the capacity of the immune system to eliminate cancer cells. Increasing these AA levels may facilitate cancer cell eradication by the immune system, but may also promote cancer growth.

Several years ago, we proposed a new approach to manipulating AA levels for cancer therapy. Instead of reducing the levels of a particular AA, we proposed the use of artificial diets to create massive changes in AA levels and ratios in order to generate toxic metabolic environments for cancer cells. The therapy would consist of replacing over several weeks the normal diet of cancer patients with an artificial diet prepared in the laboratory from scratch [54]. The rationale for this approach is as follows. Although cancer cells have mutations (and other DNA changes) that provide them with a survival advantage in a standard physiological environment, these same mutations may cause their death in a different environment. It is well known that evolution and survival depend not only on the acquisition of beneficial mutations, but also on favorable environments for these mutations. A mutation that facilitates survival in a specific environment can be lethal in a different environment. All cancer cells have originated and evolved under metabolic environments shaped by normal diets, which provide the 20 proteinogenic AAs at relatively constant levels and ratios. Creating massive changes in AA levels and ratios with artificial diets may therefore create new and unfavorable metabolic environments for cancer cells. Under these new environments, the DNA aberrations of cancer cells may cause their death [54]. Based on this idea, we evaluated the therapeutic potential of numerous artificial diets in mice with different types of metastatic cancers [26,27,119,343]. As discussed in the previous section, some of the diets induced higher survival rates in mice with metastatic cancer than those achieved with the standard pharmacological treatments, and one of the diets is currently being tested (in monotherapy) in patients with different types of metastatic cancers.

Our in vivo studies in mice with disseminate cancers revealed important conclusions that may be useful for further developing this anticancer strategy. It is generally assumed that the presence or absence of a particular AA determines the anticancer activity. However, the screening of numerous artificial diets under similar experimental conditions revealed that the restriction of a particular AA could have a positive, negative, or neutral effect on the activity of a diet, depending on the levels of its other dietary components [27]. For example, in mice with disseminated renal cell carcinomas, Cys supplementation reduced the high survival rates achieved with the most active diet formulated with free AAs (diet T2), therefore suggesting that Cys restriction was important for its activity. However, supplementing Cys in an inactive casein-based diet deficient in Cys markedly improved the survival rates of mice with the same cancer type under the same experimental conditions [27]. In mice with metastatic colon cancer, Cys supplementation markedly reduced the activity of two active diets [26]; however, in mice with peritoneally disseminated ovarian cancer, Cys supplementation did not have a significant impact on the anticancer activity of the diets [119]. We observed a similar complexity for Met. Several diets formulated with free AAs that contained relatively high amounts of Met induced a marked antitumor effect in mice with renal cell carcinomas; however, supplementing similar levels of Met in a different set of casein-based diets completely abolished their anticancer activity in the same cancer model [27]. In mice with metastatic colon cancer, Met supplementation strongly reduced the anticancer activity of two diets (diets B1 and B2B) [119]. In contrast, Met supplementation did not significantly reduce the activity of a B2B diet in mice with ovarian cancer [119]. Altogether, our results strongly suggest that changing the levels of a particular AA can have a positive, negative, or neutral effect on the anticancer activity of a diet, depending not only on the type of cancer, but also on the levels of other AAs and nutrients. The whole composition is what determines the activity.

The mechanisms by which the dietary manipulation of AAs induces selective anticancer activity are not completely understood. Unlike normal cells, cancer cells may have mutations or other DNA defects in the metabolic pathways involved in the synthesis of NEAAs, and may be unable to obtain sufficient levels if these AAs are removed from the diet [54]. In addition, since cancer cells produce higher levels of ROS than normal cells, they may have a higher dependency on Cys (and its precursor Met) to maintain sufficient GSH levels and prevent ROS-induced cell death. Another possible reason is that cancer cells proliferate faster than most normal cells and therefore need higher levels of AAs to produce the building blocks for the division of cells. Dietary AA restriction would restrict the biomass production for cancer cell division and tumor growth, while non-dividing normal cells would be unaffected. In addition, normal cells have functional checkpoints and may exit the cell cycle into a quiescent state under the conditions of AA deprivation, while cancer cells with DNA defects in the cell cycle checkpoint machinery may fail to enter quiescence. If a cell cannot stop dividing under the conditions of nutrient deprivation, the lack of specific nutrients may trigger cell death [382]. Finally, as discussed previously, the DNA aberrations that provide cancer cells with a survival advantage under normal metabolic environments may become lethal under unfavorable metabolic environments created through the dietary manipulation of AAs. Normal cells have functional DNA and would resist these temporal AA imbalances. Importantly, these unfavorable environments can be created with different types of diets, and a new metabolic environment may be toxic to cancer cells with different sets of mutations [54]. This would explain why different diets induce antitumor activity in the same cancer type, and why the same diet can be active in different types of cancer [343].

Although our review is focused on the dietary manipulation of AAs, it is important to note that the anticancer efficacy of this therapeutic strategy can be increased by simultaneously manipulating other dietary constituents. In fact, many of our active diets in mice with metastatic cancers were formulated with very low levels of lipids (1%) [26,27,119,343]. Currently, we are also manipulating other dietary constituents, such as vitamins and minerals. Since artificial diets can be prepared from scratch, any dietary component can be completely eliminated. In addition, although the dietary manipulation of AAs and other nutrients may be clinically useful as a monotherapy, future studies should evaluate artificial diets in combination with the standard therapies used for cancer patients. Although diet can have a major impact on cancer progression and responses to treatment, cancer patients are currently allowed to choose any type of diet. Future research will probably show that patient survival can be improved by matching specific therapies with specific and controlled diets.

## 7. Conclusions

Cancer cells reprogram their metabolism to produce the large amounts of building blocks required for biosynthesis and proliferation, fulfill their high energy demands, and survive under conditions of elevated oxidative stress. The altered AA metabolism of cancer cells is one of most therapeutically relevant metabolic features of cancer. In this work, we reviewed the studies that have evaluated the cancer therapeutic potential of dietary AA manipulation in vivo. These studies suggest that the dietary manipulation of AAs with artificial diets is a feasible strategy for creating harmful metabolic environments for cancer cells and achieving anticancer activity in vivo.

## 8. Patents

J.J. Jiménez-Alonso and M. López-Lázaro are the inventors of a patent related to this work licensed to AMINOVITA, S.L., and the University of Seville.

## Figures and Tables

**Table 1:** Effect of manipulating dietary protein intake in mice with cancer.

Articles	Relevant Results in Preclinical In Vivo Cancer Models
Levine et al., 2014 [31]	Low-protein diet (4% vs. 18% kcal protein) reduced IFG-1 levels and decreased tumor growth in syngeneic models of melanoma and breast cancer. Weight loss was observed in older mice.
Brandhorst et al., 2013 [32]	Low-protein diet (4% vs. 19% kcal protein) did not reduce cancer progression in a syngeneic glioma murine model.
Rubio-Patiño et al., 2018 [33]	Low-protein diet (15–17% vs. 19.5% protein) reduced cancer progression in syngeneic models of lymphoma and colon cancer. Low-protein diet enhanced anticancer immunity.
Orillion et al., 2018 [34]	Low-protein diet (7% vs. 21% protein) reduced tumor growth in syngeneic models of prostate and renal cancer by increasing antitumor immunity. Synergistic effect with immunotherapies.
Fontana et al., 2013 [35]	Low-protein diet (7% vs. 20% protein) reduced tumor growth in mice xenografts of prostate and breast cancer. Diet with a 20% plant protein showed lower tumor growth than diet with 20% animal protein.
Taha et al., 2018 [36]	Plant-based protein diet induced tumor growth inhibition compared to animal-based protein diet in two syngeneic models of ovarian cancer (20% protein in both diets).

**Table 3:** In vivo anticancer effects of Met restriction.

Articles	Relevant Results in Preclinical In Vivo Cancer Models
Sugimura et al., 1959 [39]	Dietary Met depletion (0%) for 11 days suppressed tumor growth in Walker cancer-bearing rats, causing the animals to lose l–2 g weight/day.
Goseki et al., 1991 [121]	Restriction of Met and Cys in total parenteral diet inhibited tumor growth, reduced the number of metastases, and improved survival in a sarcoma rat model. Synergistic effect with 5-fluorouracil.
Goseki et al., 1996 [122]	Restriction of Met and Cys in total parenteral diet followed by vincristine improved survival of rats with sarcoma.
Guo et al., 1996 [123]	Dietary Met depletion (0%) plus ethionine (Met analogue and antagonist) showed synergic anticancer activity in a sarcoma rat cancer model.
Xiao et al., 2001 [124]	Restriction of Met in total parenteral diet for 7 days suppressed cancer growth and prolonged survival of rats with gastric cancer. Synergistic effect with 5-fluorouracil.
Hoshiya et al., 1995 [125]	Met-free diet inhibited growth of human cancer xenografts in nude mice.
Hoshiya et al., 1996 [105]	Dietary Met depletion (0%) induced anticancer activity in mice xenografted with human breast cancer cells and increased the antitumor activity of cisplatin.
Hoshiya et al., 1997 [106]	Dietary Met depletion (0%) induced anticancer activity in mice xenografted with human gastric cancer cells and increased the antitumor activity of 5-fluorouracil.
Guo et al., 1993 [107]	Met-free diet extended mice survival in a xenograft sarcoma model, with eventual cancer regression.
Jeon et al., 2016 [108]	Met-free diet for 10 days decreased the number of tumors in the lungs in a syngeneic triple-negative breast cancer model in mice. This diet induced significant weight loss. Mice survival was not evaluated.
Strekalova et al., 2015 [109]	Met-free diet for 5 weeks inhibited tumor growth in mice xenografted with human triple-negative breast cancer cells. Synergistic effect with lexatumumab (TNF receptor agonist).
Malin et al., 2021 [110]	Met-free diet for 4 weeks showed anticancer activity against triple-negative breast cancer xenograft and PDX models. Synergistic effect with auranofin (TXNRDs inhibitor).
Lui et al., 2015 [111]	Double Cys and Met deprivation inhibited tumor growth and triggered autophagy in a xenograft glioma model in mice.
Breillout et al., 1987 [129]	Met-restricted diet supplemented with homocysteine reduced the metastatic dissemination of cancer cells in a rhabdomyosarcoma rat model.
Orillion et al., 2018 [34]	Dietary Met restriction (0.092%) induced anticancer activity in models of prostate (RP-B6Myc) and renal (RENCA) cell carcinoma. Synergistic effect with immunotherapies.
Theuer 1971 [40]	Dietary Met restriction (0.10%) for 3 weeks showed anticancer activity in a spontaneous breast adenocarcinoma model. Diets with higher Met levels (0.20–0.60%) showed no anticancer activity. All diets were also restricted in Cys.
Sinha et al., 2014 [112]	Dietary Met restriction (0.12%) for 11 weeks reduced the development and severity of prostate cancer in a transgenic murine model of prostate adenocarcinoma.
Hens et al., 2016 [115]	Dietary Met restriction (0.12%) for 12 weeks induced anticancer activity in mice xenografted with human breast cancer cells. Reduced plasma levels of Met, Cys, and Tau were reported.
Gao et al., 2019 [114]	Dietary Met restriction (0.12%) induced anticancer activity in a transgenic sarcoma and patient-derived xenograft colorectal cancer models. The diet increased the anticancer effect of radiotherapy and 5-fluorouracil.
Liu et al., 2022 [117]	Dietary Met restriction (0.12%) induced anticancer activity in mice xenografted with human colorectal cancer cells. Synergistic effect with 5-fluorouracil.
Li et al., 2023 [118]	Dietary Met restriction (0.12%) improved antitumor immunity and showed a synergistic effect with anti-PD-1 immunotherapy in two syngeneic models of colorectal cancer.
Upadhyayula et al., 2023 [120]	Dietary restriction of Cys and Met (0.0% Cys 0.15% Met vs. 0.40% Cys 0.43% Met) for 7 days induced anticancer activity in a murine glioma model. Synergistic effect with GPX4 inhibitor.
Xu et al., 2020 [113]	Dietary Met restriction (0.17%) inhibited HNF4α-positive liver cancer growth in mice
Komninou et al., 2006 [116]	Met limited diet (0.17%) inhibited the development and proliferation of colonic tumors in an induced colon cancer rat model.
Calderón-Montaño et al., 2022 [27]	Artificial diets with manipulated levels of AAs markedly improved survival of mice with disseminated renal cell carcinomas. Several active diets formulated with free AAs contained 0.60% Met. However, the anticancer activity of casein-based diets (0.17% Met) was completely blocked by adding 0.5% Met supplement.
Jiménez-Alonso et al., 2023 [119]	Artificial diets restricted in Met (0.17%) showed anticancer activity in mice with metastatic colon cancer, ovarian cancer, and renal cell carcinoma. Met supplementation blocked the anticancer activity in mice with colon cancer.

**Table 4:** In vivo anticancer effects of Cys restriction.

Articles	Relevant Results in Preclinical Cancer In Vivo Models
Voegtlin et al., 1936 [133]	A diet deficient in Cys and Met (approximately 0.06% Cys and 0.17% Met) reduced tumor growth in mice with spontaneous breast adenocarcinomas. Addition of 0.6% CysS stimulated tumor growth abruptly.
Theuer 1971 [40]	3 weeks of treatment with a diet restricted in Cys and limited in Met (0.10%) showed anticancer activity in a spontaneous breast adenocarcinoma model. Diets restricted in Cys but with higher Met levels (0.20–0.60%) showed no anticancer activity.
Zhang et al., 2020 [174]	Dietary Cys restriction reduced tumor growth in mice xenografted with human colon cancer cells. Loss/inhibition of MTAP upregulated polyamine metabolism and increased the activity of Cys restriction.
Wu et al., 2021 [175]	Dietary Cys restriction suppressed cancer growth in mice xenografted with human colon cancer cells without causing weight loss. Synergistic effect with oxaliplatin.
Ruiz-Rodado et al., 2022 [176]	Dietary Cys restriction reduced plasma levels of Cys and GSH and increased mice survival in a xenograft orthotopic glioma model.
Goseki et al., 1991 [121]	Total parenteral diet without Cys and Met induced anticancer activity in a sarcoma rat model. Synergistic effect with 5-fluorouracil.
Goseki et al., 1996 [122]	Total parenteral diet without Cys and Met followed by vincristine improved survival in a sarcoma rat cancer model.
Lui et al., 2015 [111]	Dietary depletion of Cys and Met inhibited tumor growth and triggered autophagy in a xenograft glioma model in mice.
Orillion et al., 2018 [34]	Diet restricted in Cys and limited in Met (0.092%) showed anticancer activity in a transgenic prostate cancer model in mice. Synergistic effect with immunotherapies.
Upadhyayula et al., 2023 [120]	Dietary restriction of Cys and Met (0.0% Cys 0.15% Met vs. 0.40% Cys 0.43% Met) for 7 days induced anticancer activity in a murine glioma model. Synergistic effect with GPX4 inhibitor.
Jiménez-Alonso et al., 2022 [26]	Two artificial diets deficient in Cys/Met improved survival of mice with metastatic colon cancer. The addition of 0.2% Cys blocked the anticancer activity of both diets.
Calderón-Montaño et al., 2022 [27]	Artificial diet lacking 6 NEAAs (including Cys) with normal Met levels (0.6%) showed marked anticancer activity in mice with disseminated renal cell carcinomas; the anticancer activity of this diet was reduced by supplementing Cys. However, supplementing 0.2% Cys in an inactive casein-based diet markedly improved its anticancer activity in mice with disseminated renal cell carcinoma.

**Table 5:** In vivo anticancer effects of Ser and Gly restriction.

Articles	Relevant Results in Preclinical Cancer In Vivo Models
Maddocks et al., 2013 [214] ^1^	Dietary Ser/Gly restriction induced anticancer activity in mice xenografted with p53-defective colon cancer cells.
Maddocks et al., 2017 [208] ^1^	Dietary Ser/Gly restriction induced in vivo anticancer activity that could be improved by antagonizing the anti-oxidant response
Humpton et al., 2018 [215] ^1^	A commonly occurring p53 mutant, R248W, retains wild-type ability to support survival under serine starvation. The growth of R248W-expressing tumors was resistant to dietary Ser/Gly restriction.
LeBoeuf et al., 2020 [216] ^1^	Dietary Ser/Gly restriction inhibited tumor growth in mice with mutated KEAP1. Synergistic effect with a GLS inhibitor (CB-839) even in non-mutated cancers.
Tajan et al., 2021 [217] ^1^	Dietary Ser/Gly restriction plus PH755 (PHGDH inhibitor) synergistically improved the anticancer activity in colon cancer xenografts
Falcone et al., 2022 [218] ^1^	Dietary Ser/Gly restriction improved the anticancer effect of radiotherapy in syngeneic models of pancreatic cancer and triple-negative breast cancer.
Pranzini et al., 2022 [219] ^1^	Dietary Ser/Gly restriction was inactive in syngeneic and xenograft colon cancer models. However, the combination with 5-fluorouracil showed a synergistic anticancer effect.
Gravel et al., 2014 [209]	Dietary Ser/Gly restriction reduced Ser levels in plasma and tumors. The combination of Ser–Gly-restricted diet plus phenformin reduced tumor growth in a syngeneic colon cancer model.
Polet et al., 2016 [220]	Dietary Ser/Gly restriction improved survival in a murine syngeneic model of leukemia. Combination with a GLS inhibitor (BPTES) synergistically improved mice survival.
Méndez-Lucas et al., 2020 [221]	Dietary Ser/Gly restriction plus inhibition of Ser biosynthesis (PSAT1 knockdown) showed anticancer activity in a murine model of c-MYC-induced liver cancer. Each intervention alone did not show activity.
Van Nyen et al., 2022 [210]	Dietary Ser/Gly restriction reduced tumor growth in a platinum-resistant ovarian cancer model in mice. Mice with platinum-sensitive ovarian cancer cells were insensitive to the diet.
Sullivan et al., 2019 [211]	Dietary Ser/Gly restriction plus PHGDH knockdown significantly reduced tumor growth in triple-negative breast cancer xenografts. PHGDH overexpression reduced the effect of this restriction.
Muthusamy et al., 2020 [212]	Dietary Ser/Gly restriction altered the biosynthesis of sphingolipids and decreased tumor growth in a xenograft model of colon cancer.
Fujihara et al., 2022 [213]	Dietary Ser/Gly restriction induced anticancer activity in a xenograft model of esophageal cancer. Combination with a ferroptosis inducer (eprenetapopt) synergistically improved mice survival.
Calderón-Montaño et al., 2022 [27]	An artificial diet lacking Ser and other 5 NEAAs (Ser, Cys, Tyr, Pro, Asn, and Glu) markedly improved the survival of mice with disseminated renal cell carcinoma. Ser supplementation did not decrease the activity of the diet and Gly elimination did not improve the activity of the diet.

^1^ The control diet and the Ser–Gly restricted diet both lacked the NEAAs Ala, Pro, Glu, Asn, and Ast (the only difference was the addition/restriction of Ser and Gly).

**Table 6:** In vivo anticancer effect of Arg dietary restriction or supplementation.

Articles	Relevant Results in Preclinical Cancer In Vivo Models
Gonzalez and Byus 1991 [232]	Dietary Arg restriction reduced the ornithine available for polyamine biosynthesis and reduced the incidence and multiplicity of papillomas in a mouse model of skin cancer.
Yeatman et al., 1991 [233]	Dietary Arg restriction inhibited tumor growth in a syngeneic model of colon cancer in mice. Arg supplementation stimulated tumor growth.
Alexandrou et al., 2018 [235]	Dietary Arg restriction reduced tumor growth in mice xenografted with human colorectal cancer cells deficient in ASS1 and OTC (ornithine transcarbamylase).
Cheng et al., 2018 [234]	Dietary Arg restriction reduced tumor size in two xenograft breast cancer models of ASS1-deficient cells. No weight loss was observed in the animals.
Hsu et al., 2021 [236]	Dietary Arg restriction suppressed prostate cancer growth in xenograft models. The Arg-free diet reduced cancer cell proliferation and enhanced inflammatory response.
Missiaen et al., 2022 [237]	Dietary Arg restriction induced anticancer activity in xenograft model of hepatic cancer. Combination with GNC2 and BCL2 inhibitors synergistically enhanced the anticancer response.
Cao, Feng et al., 2016 [238]	Arg supplementation (1.5 g/kg) reduced tumor growth in a syngeneic orthotopic breast cancer model. Reduction in myeloid-derived suppressor cells (MDSCs) and enhanced antitumor immune responses were observed.
Cao, Wang et al., 2016 [239]	Arg supplementation (1.5 g/kg) plus docetaxel synergistically inhibited tumor growth in a syngeneic breast cancer model. Reduction in MDSCs and enhanced antitumor immune responses were observed.
Geiger et al., 2016 [240]	Arg supplementation (1.5 g/kg) suppressed tumor growth and enhanced survival of mice with an immunogenic melanoma.
Satoh et al., 2020 [241]	Arg supplementation (1.5 g/kg) combined with cyclophosphamide and anti-PD-1 significantly increased the number of cured mice in a syngeneic colon cancer model.
He et al., 2017 [242]	Arg supplementation (2.0 g/kg) combined with anti-PD-1 immunotherapy synergistically increased the survival of mice with osteosarcoma.

## Data Availability

Not applicable.
